# Supplementary material for: Overlapping cell population expression profiling and regulatory inference in C. elegans
Source: BMC Genomics. 2016 Feb 29;17:159. doi: 10.1186/s12864-016-2482-z (PMC4772325; doi:10.1186/s12864-016-2482-z)
Supplement: Additional file 13: — Web supplement. (DOC 21 kb) [file 12864_2016_2482_MOESM13_ESM.zip › sortWeb/clusters/hier.300.clusters/284.html]

Cluster 284 

## Cluster 284

### Expression

| cnd-1 rep. 1 | cnd-1 rep. 2 | cnd-1 rep. 3 | pha-4 rep. 1 | pha-4 rep. 2 | pha-4 rep. 3 | ceh-27 | ceh-36 | ceh-6 | F21D5.9 | mir-57 | mls-2 | pal-1 | pros-1 | ttx-3 | unc-130 | hlh-16 | irx-1 | ceh-6 (+) hlh-16 (+) | ceh-6 (+) hlh-16 (-) | ceh-6 (-) hlh-16 (+) | cnd-1 singlets | pha-4 singlets | 0 | 60 | 120 | 150 | 180 | 240 | 330 | 390 | 420 | 480 | 540 | 570 | 600 | 630 | 660 | NAME | Functional description |
| --- | --- | --- | --- | --- | --- | --- | --- | --- | --- | --- | --- | --- | --- | --- | --- | --- | --- | --- | --- | --- | --- | --- | --- | --- | --- | --- | --- | --- | --- | --- | --- | --- | --- | --- | --- | --- | --- | --- | --- |
|  |  |  |  |  |  |  |  |  |  |  |  |  |  |  |  |  |  |  |  |  |  |  |  |  |  |  |  |  |  |  |  |  |  |  |  |  |  | *pkc-1* | Protein Kinase C |
|  |  |  |  |  |  |  |  |  |  |  |  |  |  |  |  |  |  |  |  |  |  |  |  |  |  |  |  |  |  |  |  |  |  |  |  |  |  | *mct-4* | MonoCarboxylate Transporter family |
|  |  |  |  |  |  |  |  |  |  |  |  |  |  |  |  |  |  |  |  |  |  |  |  |  |  |  |  |  |  |  |  |  |  |  |  |  |  | *cdgs-1* | Cdp-DaG Synthetase |
|  |  |  |  |  |  |  |  |  |  |  |  |  |  |  |  |  |  |  |  |  |  |  |  |  |  |  |  |  |  |  |  |  |  |  |  |  |  | T19D2.2 |  |
|  |  |  |  |  |  |  |  |  |  |  |  |  |  |  |  |  |  |  |  |  |  |  |  |  |  |  |  |  |  |  |  |  |  |  |  |  |  | T28F3.4 |  |
|  |  |  |  |  |  |  |  |  |  |  |  |  |  |  |  |  |  |  |  |  |  |  |  |  |  |  |  |  |  |  |  |  |  |  |  |  |  | *gly-20* | GLYcosylation related |
|  |  |  |  |  |  |  |  |  |  |  |  |  |  |  |  |  |  |  |  |  |  |  |  |  |  |  |  |  |  |  |  |  |  |  |  |  |  | *klp-3* | Kinesin-Like Protein |
|  |  |  |  |  |  |  |  |  |  |  |  |  |  |  |  |  |  |  |  |  |  |  |  |  |  |  |  |  |  |  |  |  |  |  |  |  |  | *tbc-7* | TBC (Tre-2/Bub2/Cdc16) domain family |
|  |  |  |  |  |  |  |  |  |  |  |  |  |  |  |  |  |  |  |  |  |  |  |  |  |  |  |  |  |  |  |  |  |  |  |  |  |  | Y106G6G.2 |  |
|  |  |  |  |  |  |  |  |  |  |  |  |  |  |  |  |  |  |  |  |  |  |  |  |  |  |  |  |  |  |  |  |  |  |  |  |  |  | *unc-31* | UNCoordinated |
|  |  |  |  |  |  |  |  |  |  |  |  |  |  |  |  |  |  |  |  |  |  |  |  |  |  |  |  |  |  |  |  |  |  |  |  |  |  | Y106G6G.4 |  |
|  |  |  |  |  |  |  |  |  |  |  |  |  |  |  |  |  |  |  |  |  |  |  |  |  |  |  |  |  |  |  |  |  |  |  |  |  |  | *fbxc-32* | F-box C protein |
|  |  |  |  |  |  |  |  |  |  |  |  |  |  |  |  |  |  |  |  |  |  |  |  |  |  |  |  |  |  |  |  |  |  |  |  |  |  | *pitp-1* | PhosphatidylInositol Transfer Protein family |
|  |  |  |  |  |  |  |  |  |  |  |  |  |  |  |  |  |  |  |  |  |  |  |  |  |  |  |  |  |  |  |  |  |  |  |  |  |  | R13A5.9 |  |
|  |  |  |  |  |  |  |  |  |  |  |  |  |  |  |  |  |  |  |  |  |  |  |  |  |  |  |  |  |  |  |  |  |  |  |  |  |  | *rskn-1* | RSK-pNinety (RSK-p90 kinase) homolog |
|  |  |  |  |  |  |  |  |  |  |  |  |  |  |  |  |  |  |  |  |  |  |  |  |  |  |  |  |  |  |  |  |  |  |  |  |  |  | *ida-1* | related to Islet cell Diabetes Autoantigen |
|  |  |  |  |  |  |  |  |  |  |  |  |  |  |  |  |  |  |  |  |  |  |  |  |  |  |  |  |  |  |  |  |  |  |  |  |  |  | *ctbp-1* | CTBP (CtBP) transcriptional co-repressor homolog |
|  |  |  |  |  |  |  |  |  |  |  |  |  |  |  |  |  |  |  |  |  |  |  |  |  |  |  |  |  |  |  |  |  |  |  |  |  |  | *casy-1* | CAlSYntenin/Alcadein homolog |
|  |  |  |  |  |  |  |  |  |  |  |  |  |  |  |  |  |  |  |  |  |  |  |  |  |  |  |  |  |  |  |  |  |  |  |  |  |  | *syd-1* | SYnapse Defective |
|  |  |  |  |  |  |  |  |  |  |  |  |  |  |  |  |  |  |  |  |  |  |  |  |  |  |  |  |  |  |  |  |  |  |  |  |  |  | F21D12.3 |  |
|  |  |  |  |  |  |  |  |  |  |  |  |  |  |  |  |  |  |  |  |  |  |  |  |  |  |  |  |  |  |  |  |  |  |  |  |  |  | T14B1.1 |  |
|  |  |  |  |  |  |  |  |  |  |  |  |  |  |  |  |  |  |  |  |  |  |  |  |  |  |  |  |  |  |  |  |  |  |  |  |  |  | *mrp-6* | Multidrug Resistance Protein family |
|  |  |  |  |  |  |  |  |  |  |  |  |  |  |  |  |  |  |  |  |  |  |  |  |  |  |  |  |  |  |  |  |  |  |  |  |  |  | *cmk-1* | CaM Kinase |
|  |  |  |  |  |  |  |  |  |  |  |  |  |  |  |  |  |  |  |  |  |  |  |  |  |  |  |  |  |  |  |  |  |  |  |  |  |  | *unc-2* | UNCoordinated |
|  |  |  |  |  |  |  |  |  |  |  |  |  |  |  |  |  |  |  |  |  |  |  |  |  |  |  |  |  |  |  |  |  |  |  |  |  |  | H01G02.3 |  |
|  |  |  |  |  |  |  |  |  |  |  |  |  |  |  |  |  |  |  |  |  |  |  |  |  |  |  |  |  |  |  |  |  |  |  |  |  |  | *egl-21* | EGg Laying defective |
|  |  |  |  |  |  |  |  |  |  |  |  |  |  |  |  |  |  |  |  |  |  |  |  |  |  |  |  |  |  |  |  |  |  |  |  |  |  | K09E9.1 |  |
|  |  |  |  |  |  |  |  |  |  |  |  |  |  |  |  |  |  |  |  |  |  |  |  |  |  |  |  |  |  |  |  |  |  |  |  |  |  | *adm-2* | ADAM (disintegrin plus metalloprotease) family |
|  |  |  |  |  |  |  |  |  |  |  |  |  |  |  |  |  |  |  |  |  |  |  |  |  |  |  |  |  |  |  |  |  |  |  |  |  |  | *ptc-1* | PaTChed family |
|  |  |  |  |  |  |  |  |  |  |  |  |  |  |  |  |  |  |  |  |  |  |  |  |  |  |  |  |  |  |  |  |  |  |  |  |  |  | Y54G2A.26 |  |
|  |  |  |  |  |  |  |  |  |  |  |  |  |  |  |  |  |  |  |  |  |  |  |  |  |  |  |  |  |  |  |  |  |  |  |  |  |  | *dgk-4* | DiacylGlycerol Kinase |
|  |  |  |  |  |  |  |  |  |  |  |  |  |  |  |  |  |  |  |  |  |  |  |  |  |  |  |  |  |  |  |  |  |  |  |  |  |  | *unc-36* | UNCoordinated |
|  |  |  |  |  |  |  |  |  |  |  |  |  |  |  |  |  |  |  |  |  |  |  |  |  |  |  |  |  |  |  |  |  |  |  |  |  |  | F35H12.1 |  |
|  |  |  |  |  |  |  |  |  |  |  |  |  |  |  |  |  |  |  |  |  |  |  |  |  |  |  |  |  |  |  |  |  |  |  |  |  |  | *egl-3* | EGg Laying defective |
|  |  |  |  |  |  |  |  |  |  |  |  |  |  |  |  |  |  |  |  |  |  |  |  |  |  |  |  |  |  |  |  |  |  |  |  |  |  | *apt-9* | AdaPTin or adaptin-related protein |
|  |  |  |  |  |  |  |  |  |  |  |  |  |  |  |  |  |  |  |  |  |  |  |  |  |  |  |  |  |  |  |  |  |  |  |  |  |  | R07E4.1 |  |
|  |  |  |  |  |  |  |  |  |  |  |  |  |  |  |  |  |  |  |  |  |  |  |  |  |  |  |  |  |  |  |  |  |  |  |  |  |  | *twk-13* | TWiK family of potassium channels |
|  |  |  |  |  |  |  |  |  |  |  |  |  |  |  |  |  |  |  |  |  |  |  |  |  |  |  |  |  |  |  |  |  |  |  |  |  |  | *frpr-18* | FMRFamide Peptide Receptor family |
|  |  |  |  |  |  |  |  |  |  |  |  |  |  |  |  |  |  |  |  |  |  |  |  |  |  |  |  |  |  |  |  |  |  |  |  |  |  | *sue-1* | SUppressor of Elongation defect |
|  |  |  |  |  |  |  |  |  |  |  |  |  |  |  |  |  |  |  |  |  |  |  |  |  |  |  |  |  |  |  |  |  |  |  |  |  |  | *ptb-1* | human PTB (hnRNP) homolog |
|  |  |  |  |  |  |  |  |  |  |  |  |  |  |  |  |  |  |  |  |  |  |  |  |  |  |  |  |  |  |  |  |  |  |  |  |  |  | *gdi-1* | GDI (RabGDP Dissociation Inhibitor) family |
|  |  |  |  |  |  |  |  |  |  |  |  |  |  |  |  |  |  |  |  |  |  |  |  |  |  |  |  |  |  |  |  |  |  |  |  |  |  | *ptr-5* | PaTched Related family |
|  |  |  |  |  |  |  |  |  |  |  |  |  |  |  |  |  |  |  |  |  |  |  |  |  |  |  |  |  |  |  |  |  |  |  |  |  |  | F35H12.7 |  |
|  |  |  |  |  |  |  |  |  |  |  |  |  |  |  |  |  |  |  |  |  |  |  |  |  |  |  |  |  |  |  |  |  |  |  |  |  |  | *tag-97* | Temporarily Assigned Gene name |
|  |  |  |  |  |  |  |  |  |  |  |  |  |  |  |  |  |  |  |  |  |  |  |  |  |  |  |  |  |  |  |  |  |  |  |  |  |  | *npr-23* | NeuroPeptide Receptor family |
|  |  |  |  |  |  |  |  |  |  |  |  |  |  |  |  |  |  |  |  |  |  |  |  |  |  |  |  |  |  |  |  |  |  |  |  |  |  | *egl-8* | EGg Laying defective |
|  |  |  |  |  |  |  |  |  |  |  |  |  |  |  |  |  |  |  |  |  |  |  |  |  |  |  |  |  |  |  |  |  |  |  |  |  |  | *frpr-13* | FMRFamide Peptide Receptor family |
|  |  |  |  |  |  |  |  |  |  |  |  |  |  |  |  |  |  |  |  |  |  |  |  |  |  |  |  |  |  |  |  |  |  |  |  |  |  | *pde-3* | PhosphoDiEsterase |
|  |  |  |  |  |  |  |  |  |  |  |  |  |  |  |  |  |  |  |  |  |  |  |  |  |  |  |  |  |  |  |  |  |  |  |  |  |  | *unc-103* | UNCoordinated |
|  |  |  |  |  |  |  |  |  |  |  |  |  |  |  |  |  |  |  |  |  |  |  |  |  |  |  |  |  |  |  |  |  |  |  |  |  |  | C34F11.3 |  |
|  |  |  |  |  |  |  |  |  |  |  |  |  |  |  |  |  |  |  |  |  |  |  |  |  |  |  |  |  |  |  |  |  |  |  |  |  |  | *daf-4* | abnormal DAuer Formation |
|  |  |  |  |  |  |  |  |  |  |  |  |  |  |  |  |  |  |  |  |  |  |  |  |  |  |  |  |  |  |  |  |  |  |  |  |  |  | T23F2.2 |  |
|  |  |  |  |  |  |  |  |  |  |  |  |  |  |  |  |  |  |  |  |  |  |  |  |  |  |  |  |  |  |  |  |  |  |  |  |  |  | *pme-5* | Poly(ADP-ribose) Metabolism Enzyme |
|  |  |  |  |  |  |  |  |  |  |  |  |  |  |  |  |  |  |  |  |  |  |  |  |  |  |  |  |  |  |  |  |  |  |  |  |  |  | *fbl-1* | FiBuLin |
|  |  |  |  |  |  |  |  |  |  |  |  |  |  |  |  |  |  |  |  |  |  |  |  |  |  |  |  |  |  |  |  |  |  |  |  |  |  | H11E01.3 |  |
|  |  |  |  |  |  |  |  |  |  |  |  |  |  |  |  |  |  |  |  |  |  |  |  |  |  |  |  |  |  |  |  |  |  |  |  |  |  | Y51A2D.1 |  |
|  |  |  |  |  |  |  |  |  |  |  |  |  |  |  |  |  |  |  |  |  |  |  |  |  |  |  |  |  |  |  |  |  |  |  |  |  |  | Y97E10AR.8 |  |
|  |  |  |  |  |  |  |  |  |  |  |  |  |  |  |  |  |  |  |  |  |  |  |  |  |  |  |  |  |  |  |  |  |  |  |  |  |  | *vha-15* | Vacuolar H ATPase |
|  |  |  |  |  |  |  |  |  |  |  |  |  |  |  |  |  |  |  |  |  |  |  |  |  |  |  |  |  |  |  |  |  |  |  |  |  |  | *mgl-1* | Metabotropic GLutamate receptor family |
|  |  |  |  |  |  |  |  |  |  |  |  |  |  |  |  |  |  |  |  |  |  |  |  |  |  |  |  |  |  |  |  |  |  |  |  |  |  | *pdf-1* | PDF (arthropod Pigment Dispersing Factor) homolog |
|  |  |  |  |  |  |  |  |  |  |  |  |  |  |  |  |  |  |  |  |  |  |  |  |  |  |  |  |  |  |  |  |  |  |  |  |  |  | *atln-2* | ATLastiN (endoplasmic reticulum GTPase) related |
|  |  |  |  |  |  |  |  |  |  |  |  |  |  |  |  |  |  |  |  |  |  |  |  |  |  |  |  |  |  |  |  |  |  |  |  |  |  | M04B2.7 |  |
|  |  |  |  |  |  |  |  |  |  |  |  |  |  |  |  |  |  |  |  |  |  |  |  |  |  |  |  |  |  |  |  |  |  |  |  |  |  | *gnrr-4* | human GoNadotropin-Releasing hormone Receptor (GnRHR) related |
|  |  |  |  |  |  |  |  |  |  |  |  |  |  |  |  |  |  |  |  |  |  |  |  |  |  |  |  |  |  |  |  |  |  |  |  |  |  | F35B12.4 |  |
|  |  |  |  |  |  |  |  |  |  |  |  |  |  |  |  |  |  |  |  |  |  |  |  |  |  |  |  |  |  |  |  |  |  |  |  |  |  | *srab-12* | Serpentine Receptor, class AB (class A-like) |
|  |  |  |  |  |  |  |  |  |  |  |  |  |  |  |  |  |  |  |  |  |  |  |  |  |  |  |  |  |  |  |  |  |  |  |  |  |  | *zip-1* | bZIP transcription factor family |
|  |  |  |  |  |  |  |  |  |  |  |  |  |  |  |  |  |  |  |  |  |  |  |  |  |  |  |  |  |  |  |  |  |  |  |  |  |  | *srsx-38* | Serpentine Receptor, class SX |
|  |  |  |  |  |  |  |  |  |  |  |  |  |  |  |  |  |  |  |  |  |  |  |  |  |  |  |  |  |  |  |  |  |  |  |  |  |  | *srz-19* | Serpentine Receptor, class Z |
|  |  |  |  |  |  |  |  |  |  |  |  |  |  |  |  |  |  |  |  |  |  |  |  |  |  |  |  |  |  |  |  |  |  |  |  |  |  | R05G6.1 |  |
|  |  |  |  |  |  |  |  |  |  |  |  |  |  |  |  |  |  |  |  |  |  |  |  |  |  |  |  |  |  |  |  |  |  |  |  |  |  | K02B12.7 |  |
|  |  |  |  |  |  |  |  |  |  |  |  |  |  |  |  |  |  |  |  |  |  |  |  |  |  |  |  |  |  |  |  |  |  |  |  |  |  | *med-2* | Mesoderm and Endoderm Determination |
|  |  |  |  |  |  |  |  |  |  |  |  |  |  |  |  |  |  |  |  |  |  |  |  |  |  |  |  |  |  |  |  |  |  |  |  |  |  | R05G6.10 |  |
|  |  |  |  |  |  |  |  |  |  |  |  |  |  |  |  |  |  |  |  |  |  |  |  |  |  |  |  |  |  |  |  |  |  |  |  |  |  | *pqn-18* | Prion-like-(Q/N-rich)-domain-bearing protein |
|  |  |  |  |  |  |  |  |  |  |  |  |  |  |  |  |  |  |  |  |  |  |  |  |  |  |  |  |  |  |  |  |  |  |  |  |  |  | *nhr-1* | Nuclear Hormone Receptor family |
|  |  |  |  |  |  |  |  |  |  |  |  |  |  |  |  |  |  |  |  |  |  |  |  |  |  |  |  |  |  |  |  |  |  |  |  |  |  | *trpp-8* | TRansport Protein Particle |
|  |  |  |  |  |  |  |  |  |  |  |  |  |  |  |  |  |  |  |  |  |  |  |  |  |  |  |  |  |  |  |  |  |  |  |  |  |  | *mlk-1* | human MLK (Mixed Lineage Kinase) homolog |
|  |  |  |  |  |  |  |  |  |  |  |  |  |  |  |  |  |  |  |  |  |  |  |  |  |  |  |  |  |  |  |  |  |  |  |  |  |  | Y97E10AR.1 |  |
|  |  |  |  |  |  |  |  |  |  |  |  |  |  |  |  |  |  |  |  |  |  |  |  |  |  |  |  |  |  |  |  |  |  |  |  |  |  | F53B1.8 |  |
|  |  |  |  |  |  |  |  |  |  |  |  |  |  |  |  |  |  |  |  |  |  |  |  |  |  |  |  |  |  |  |  |  |  |  |  |  |  | *nhr-40* | Nuclear Hormone Receptor family |
|  |  |  |  |  |  |  |  |  |  |  |  |  |  |  |  |  |  |  |  |  |  |  |  |  |  |  |  |  |  |  |  |  |  |  |  |  |  | C26B9.6 |  |
|  |  |  |  |  |  |  |  |  |  |  |  |  |  |  |  |  |  |  |  |  |  |  |  |  |  |  |  |  |  |  |  |  |  |  |  |  |  | *rskn-2* | RSK-pNinety (RSK-p90 kinase) homolog |
|  |  |  |  |  |  |  |  |  |  |  |  |  |  |  |  |  |  |  |  |  |  |  |  |  |  |  |  |  |  |  |  |  |  |  |  |  |  | K02A6.3 |  |
|  |  |  |  |  |  |  |  |  |  |  |  |  |  |  |  |  |  |  |  |  |  |  |  |  |  |  |  |  |  |  |  |  |  |  |  |  |  | M02B1.2 |  |
|  |  |  |  |  |  |  |  |  |  |  |  |  |  |  |  |  |  |  |  |  |  |  |  |  |  |  |  |  |  |  |  |  |  |  |  |  |  | *gst-11* | Glutathione S-Transferase |
|  |  |  |  |  |  |  |  |  |  |  |  |  |  |  |  |  |  |  |  |  |  |  |  |  |  |  |  |  |  |  |  |  |  |  |  |  |  | *hpo-30* | Hypersensitive to POre-forming toxin |
|  |  |  |  |  |  |  |  |  |  |  |  |  |  |  |  |  |  |  |  |  |  |  |  |  |  |  |  |  |  |  |  |  |  |  |  |  |  | *nhr-32* | Nuclear Hormone Receptor family |
|  |  |  |  |  |  |  |  |  |  |  |  |  |  |  |  |  |  |  |  |  |  |  |  |  |  |  |  |  |  |  |  |  |  |  |  |  |  | *vit-5* | VITellogenin structural genes (yolk protein genes) |
|  |  |  |  |  |  |  |  |  |  |  |  |  |  |  |  |  |  |  |  |  |  |  |  |  |  |  |  |  |  |  |  |  |  |  |  |  |  | C32D5.6 |  |
|  |  |  |  |  |  |  |  |  |  |  |  |  |  |  |  |  |  |  |  |  |  |  |  |  |  |  |  |  |  |  |  |  |  |  |  |  |  | F48E3.2 |  |
|  |  |  |  |  |  |  |  |  |  |  |  |  |  |  |  |  |  |  |  |  |  |  |  |  |  |  |  |  |  |  |  |  |  |  |  |  |  | C33D9.3 |  |
|  |  |  |  |  |  |  |  |  |  |  |  |  |  |  |  |  |  |  |  |  |  |  |  |  |  |  |  |  |  |  |  |  |  |  |  |  |  | *kvs-3* | K (potassium) Voltage-Sensitive channel subunit |
|  |  |  |  |  |  |  |  |  |  |  |  |  |  |  |  |  |  |  |  |  |  |  |  |  |  |  |  |  |  |  |  |  |  |  |  |  |  | *vps-26* | related to yeast Vacuolar Protein Sorting factor |
|  |  |  |  |  |  |  |  |  |  |  |  |  |  |  |  |  |  |  |  |  |  |  |  |  |  |  |  |  |  |  |  |  |  |  |  |  |  | B0513.5 |  |
|  |  |  |  |  |  |  |  |  |  |  |  |  |  |  |  |  |  |  |  |  |  |  |  |  |  |  |  |  |  |  |  |  |  |  |  |  |  | *rfs-1* | Rad-51 (Fifty-one) like, Short |
|  |  |  |  |  |  |  |  |  |  |  |  |  |  |  |  |  |  |  |  |  |  |  |  |  |  |  |  |  |  |  |  |  |  |  |  |  |  | C02F5.7 |  |
|  |  |  |  |  |  |  |  |  |  |  |  |  |  |  |  |  |  |  |  |  |  |  |  |  |  |  |  |  |  |  |  |  |  |  |  |  |  | B0454.8 |  |
|  |  |  |  |  |  |  |  |  |  |  |  |  |  |  |  |  |  |  |  |  |  |  |  |  |  |  |  |  |  |  |  |  |  |  |  |  |  | T12A7.6 |  |
|  |  |  |  |  |  |  |  |  |  |  |  |  |  |  |  |  |  |  |  |  |  |  |  |  |  |  |  |  |  |  |  |  |  |  |  |  |  | C32E8.9 |  |
|  |  |  |  |  |  |  |  |  |  |  |  |  |  |  |  |  |  |  |  |  |  |  |  |  |  |  |  |  |  |  |  |  |  |  |  |  |  | K09E3.6 |  |
|  |  |  |  |  |  |  |  |  |  |  |  |  |  |  |  |  |  |  |  |  |  |  |  |  |  |  |  |  |  |  |  |  |  |  |  |  |  | *gcl-1* | GCL (Drosophila Germ Cell-Less) homolog |
|  |  |  |  |  |  |  |  |  |  |  |  |  |  |  |  |  |  |  |  |  |  |  |  |  |  |  |  |  |  |  |  |  |  |  |  |  |  | *tag-68* | Temporarily Assigned Gene name |
|  |  |  |  |  |  |  |  |  |  |  |  |  |  |  |  |  |  |  |  |  |  |  |  |  |  |  |  |  |  |  |  |  |  |  |  |  |  | *mcu-1* | Mitochondrial Calcium Uniporter |
|  |  |  |  |  |  |  |  |  |  |  |  |  |  |  |  |  |  |  |  |  |  |  |  |  |  |  |  |  |  |  |  |  |  |  |  |  |  | *gnrr-5* | human GoNadotropin-Releasing hormone Receptor (GnRHR) related |
|  |  |  |  |  |  |  |  |  |  |  |  |  |  |  |  |  |  |  |  |  |  |  |  |  |  |  |  |  |  |  |  |  |  |  |  |  |  | *tts-2* | Transcribed Telomerase-like Sequence |
|  |  |  |  |  |  |  |  |  |  |  |  |  |  |  |  |  |  |  |  |  |  |  |  |  |  |  |  |  |  |  |  |  |  |  |  |  |  | Y97E10C.1 |  |
|  |  |  |  |  |  |  |  |  |  |  |  |  |  |  |  |  |  |  |  |  |  |  |  |  |  |  |  |  |  |  |  |  |  |  |  |  |  | M02F4.3 |  |
|  |  |  |  |  |  |  |  |  |  |  |  |  |  |  |  |  |  |  |  |  |  |  |  |  |  |  |  |  |  |  |  |  |  |  |  |  |  | *unc-5* | UNCoordinated |
|  |  |  |  |  |  |  |  |  |  |  |  |  |  |  |  |  |  |  |  |  |  |  |  |  |  |  |  |  |  |  |  |  |  |  |  |  |  | AH9.6 |  |
|  |  |  |  |  |  |  |  |  |  |  |  |  |  |  |  |  |  |  |  |  |  |  |  |  |  |  |  |  |  |  |  |  |  |  |  |  |  | *oct-2* | Organic Cation Transporter |
|  |  |  |  |  |  |  |  |  |  |  |  |  |  |  |  |  |  |  |  |  |  |  |  |  |  |  |  |  |  |  |  |  |  |  |  |  |  | *egl-19* | EGg Laying defective |
|  |  |  |  |  |  |  |  |  |  |  |  |  |  |  |  |  |  |  |  |  |  |  |  |  |  |  |  |  |  |  |  |  |  |  |  |  |  | DH11.5 |  |
|  |  |  |  |  |  |  |  |  |  |  |  |  |  |  |  |  |  |  |  |  |  |  |  |  |  |  |  |  |  |  |  |  |  |  |  |  |  | *unc-43* | UNCoordinated |
|  |  |  |  |  |  |  |  |  |  |  |  |  |  |  |  |  |  |  |  |  |  |  |  |  |  |  |  |  |  |  |  |  |  |  |  |  |  | *jph-1* | JunctoPHilin |
|  |  |  |  |  |  |  |  |  |  |  |  |  |  |  |  |  |  |  |  |  |  |  |  |  |  |  |  |  |  |  |  |  |  |  |  |  |  | *dop-5* | DOPamine receptor |
|  |  |  |  |  |  |  |  |  |  |  |  |  |  |  |  |  |  |  |  |  |  |  |  |  |  |  |  |  |  |  |  |  |  |  |  |  |  | T28D6.4 |  |
|  |  |  |  |  |  |  |  |  |  |  |  |  |  |  |  |  |  |  |  |  |  |  |  |  |  |  |  |  |  |  |  |  |  |  |  |  |  | R02F11.3 |  |
|  |  |  |  |  |  |  |  |  |  |  |  |  |  |  |  |  |  |  |  |  |  |  |  |  |  |  |  |  |  |  |  |  |  |  |  |  |  | *snpc-1.4* | SNAPc (Small Nuclear RNA Activating Complex) homolog |
|  |  |  |  |  |  |  |  |  |  |  |  |  |  |  |  |  |  |  |  |  |  |  |  |  |  |  |  |  |  |  |  |  |  |  |  |  |  | *lis-1* | LIS-1 (human lissencephaly gene) related |
|  |  |  |  |  |  |  |  |  |  |  |  |  |  |  |  |  |  |  |  |  |  |  |  |  |  |  |  |  |  |  |  |  |  |  |  |  |  | *myo-6* | MYOsin heavy chain structural genes |
|  |  |  |  |  |  |  |  |  |  |  |  |  |  |  |  |  |  |  |  |  |  |  |  |  |  |  |  |  |  |  |  |  |  |  |  |  |  | *twk-43* | TWiK family of potassium channels |
|  |  |  |  |  |  |  |  |  |  |  |  |  |  |  |  |  |  |  |  |  |  |  |  |  |  |  |  |  |  |  |  |  |  |  |  |  |  | *unc-94* | UNCoordinated |
|  |  |  |  |  |  |  |  |  |  |  |  |  |  |  |  |  |  |  |  |  |  |  |  |  |  |  |  |  |  |  |  |  |  |  |  |  |  | *nhr-20* | Nuclear Hormone Receptor family |
|  |  |  |  |  |  |  |  |  |  |  |  |  |  |  |  |  |  |  |  |  |  |  |  |  |  |  |  |  |  |  |  |  |  |  |  |  |  | T23G5.2 |  |
|  |  |  |  |  |  |  |  |  |  |  |  |  |  |  |  |  |  |  |  |  |  |  |  |  |  |  |  |  |  |  |  |  |  |  |  |  |  | F26A10.2 |  |
|  |  |  |  |  |  |  |  |  |  |  |  |  |  |  |  |  |  |  |  |  |  |  |  |  |  |  |  |  |  |  |  |  |  |  |  |  |  | *vhp-1* | VH1 dual-specificity phosphatase family |
|  |  |  |  |  |  |  |  |  |  |  |  |  |  |  |  |  |  |  |  |  |  |  |  |  |  |  |  |  |  |  |  |  |  |  |  |  |  | *wdfy-2* | WD40 and FYVE domain protein |
|  |  |  |  |  |  |  |  |  |  |  |  |  |  |  |  |  |  |  |  |  |  |  |  |  |  |  |  |  |  |  |  |  |  |  |  |  |  | T27A3.1 |  |
|  |  |  |  |  |  |  |  |  |  |  |  |  |  |  |  |  |  |  |  |  |  |  |  |  |  |  |  |  |  |  |  |  |  |  |  |  |  | *par-1* | abnormal embryonic PARtitioning of cytoplasm |
|  |  |  |  |  |  |  |  |  |  |  |  |  |  |  |  |  |  |  |  |  |  |  |  |  |  |  |  |  |  |  |  |  |  |  |  |  |  | *ccb-1* | Calcium Channel, Beta subunit |
|  |  |  |  |  |  |  |  |  |  |  |  |  |  |  |  |  |  |  |  |  |  |  |  |  |  |  |  |  |  |  |  |  |  |  |  |  |  | F53C11.4 |  |
|  |  |  |  |  |  |  |  |  |  |  |  |  |  |  |  |  |  |  |  |  |  |  |  |  |  |  |  |  |  |  |  |  |  |  |  |  |  | Y32G9A.5 |  |
|  |  |  |  |  |  |  |  |  |  |  |  |  |  |  |  |  |  |  |  |  |  |  |  |  |  |  |  |  |  |  |  |  |  |  |  |  |  | *atgp-1* | Amino acid Transporter GlycoProtein subunit |
|  |  |  |  |  |  |  |  |  |  |  |  |  |  |  |  |  |  |  |  |  |  |  |  |  |  |  |  |  |  |  |  |  |  |  |  |  |  | Y43F8C.3 |  |
|  |  |  |  |  |  |  |  |  |  |  |  |  |  |  |  |  |  |  |  |  |  |  |  |  |  |  |  |  |  |  |  |  |  |  |  |  |  | *kin-1* | protein KINase |
|  |  |  |  |  |  |  |  |  |  |  |  |  |  |  |  |  |  |  |  |  |  |  |  |  |  |  |  |  |  |  |  |  |  |  |  |  |  | T04B2.5 |  |
|  |  |  |  |  |  |  |  |  |  |  |  |  |  |  |  |  |  |  |  |  |  |  |  |  |  |  |  |  |  |  |  |  |  |  |  |  |  | *crh-2* | CREB Homolog |
|  |  |  |  |  |  |  |  |  |  |  |  |  |  |  |  |  |  |  |  |  |  |  |  |  |  |  |  |  |  |  |  |  |  |  |  |  |  | B0228.9 |  |
|  |  |  |  |  |  |  |  |  |  |  |  |  |  |  |  |  |  |  |  |  |  |  |  |  |  |  |  |  |  |  |  |  |  |  |  |  |  | F42H10.9 |  |
|  |  |  |  |  |  |  |  |  |  |  |  |  |  |  |  |  |  |  |  |  |  |  |  |  |  |  |  |  |  |  |  |  |  |  |  |  |  | C46H11.10 |  |
|  |  |  |  |  |  |  |  |  |  |  |  |  |  |  |  |  |  |  |  |  |  |  |  |  |  |  |  |  |  |  |  |  |  |  |  |  |  | *drap-1* | DRAP1 corepressor homolog |
|  |  |  |  |  |  |  |  |  |  |  |  |  |  |  |  |  |  |  |  |  |  |  |  |  |  |  |  |  |  |  |  |  |  |  |  |  |  | *lips-6* | LIPaSe related |
|  |  |  |  |  |  |  |  |  |  |  |  |  |  |  |  |  |  |  |  |  |  |  |  |  |  |  |  |  |  |  |  |  |  |  |  |  |  | K01D12.5 |  |
|  |  |  |  |  |  |  |  |  |  |  |  |  |  |  |  |  |  |  |  |  |  |  |  |  |  |  |  |  |  |  |  |  |  |  |  |  |  | *gab-1* | GABa receptor subunit |
|  |  |  |  |  |  |  |  |  |  |  |  |  |  |  |  |  |  |  |  |  |  |  |  |  |  |  |  |  |  |  |  |  |  |  |  |  |  | M88.3 |  |
|  |  |  |  |  |  |  |  |  |  |  |  |  |  |  |  |  |  |  |  |  |  |  |  |  |  |  |  |  |  |  |  |  |  |  |  |  |  | C34B2.9 |  |
|  |  |  |  |  |  |  |  |  |  |  |  |  |  |  |  |  |  |  |  |  |  |  |  |  |  |  |  |  |  |  |  |  |  |  |  |  |  | *set-11* | SET (trithorax/polycomb) domain containing |
|  |  |  |  |  |  |  |  |  |  |  |  |  |  |  |  |  |  |  |  |  |  |  |  |  |  |  |  |  |  |  |  |  |  |  |  |  |  | *elo-3* | fatty acid ELOngation |
|  |  |  |  |  |  |  |  |  |  |  |  |  |  |  |  |  |  |  |  |  |  |  |  |  |  |  |  |  |  |  |  |  |  |  |  |  |  | C33D12.2 |  |
|  |  |  |  |  |  |  |  |  |  |  |  |  |  |  |  |  |  |  |  |  |  |  |  |  |  |  |  |  |  |  |  |  |  |  |  |  |  | *egl-36* | EGg Laying defective |
|  |  |  |  |  |  |  |  |  |  |  |  |  |  |  |  |  |  |  |  |  |  |  |  |  |  |  |  |  |  |  |  |  |  |  |  |  |  | *cpb-3* | CPEB polyA binding family |
|  |  |  |  |  |  |  |  |  |  |  |  |  |  |  |  |  |  |  |  |  |  |  |  |  |  |  |  |  |  |  |  |  |  |  |  |  |  | C15C7.6 |  |
|  |  |  |  |  |  |  |  |  |  |  |  |  |  |  |  |  |  |  |  |  |  |  |  |  |  |  |  |  |  |  |  |  |  |  |  |  |  | *npr-33* | NeuroPeptide Receptor family |
|  |  |  |  |  |  |  |  |  |  |  |  |  |  |  |  |  |  |  |  |  |  |  |  |  |  |  |  |  |  |  |  |  |  |  |  |  |  | C16D2.3 |  |
|  |  |  |  |  |  |  |  |  |  |  |  |  |  |  |  |  |  |  |  |  |  |  |  |  |  |  |  |  |  |  |  |  |  |  |  |  |  | ZK1321.1 |  |
|  |  |  |  |  |  |  |  |  |  |  |  |  |  |  |  |  |  |  |  |  |  |  |  |  |  |  |  |  |  |  |  |  |  |  |  |  |  | K02E10.5 |  |
|  |  |  |  |  |  |  |  |  |  |  |  |  |  |  |  |  |  |  |  |  |  |  |  |  |  |  |  |  |  |  |  |  |  |  |  |  |  | F25H5.8 |  |
|  |  |  |  |  |  |  |  |  |  |  |  |  |  |  |  |  |  |  |  |  |  |  |  |  |  |  |  |  |  |  |  |  |  |  |  |  |  | T08G5.14 |  |
|  |  |  |  |  |  |  |  |  |  |  |  |  |  |  |  |  |  |  |  |  |  |  |  |  |  |  |  |  |  |  |  |  |  |  |  |  |  | C40H1.2 |  |
|  |  |  |  |  |  |  |  |  |  |  |  |  |  |  |  |  |  |  |  |  |  |  |  |  |  |  |  |  |  |  |  |  |  |  |  |  |  | F12D9.9 |  |
|  |  |  |  |  |  |  |  |  |  |  |  |  |  |  |  |  |  |  |  |  |  |  |  |  |  |  |  |  |  |  |  |  |  |  |  |  |  | F47D12.3 |  |
|  |  |  |  |  |  |  |  |  |  |  |  |  |  |  |  |  |  |  |  |  |  |  |  |  |  |  |  |  |  |  |  |  |  |  |  |  |  | F29B9.12 |  |
|  |  |  |  |  |  |  |  |  |  |  |  |  |  |  |  |  |  |  |  |  |  |  |  |  |  |  |  |  |  |  |  |  |  |  |  |  |  | B0228.1 |  |
|  |  |  |  |  |  |  |  |  |  |  |  |  |  |  |  |  |  |  |  |  |  |  |  |  |  |  |  |  |  |  |  |  |  |  |  |  |  | *hot-2* | Homolog of Odr-2 (Two) |
|  |  |  |  |  |  |  |  |  |  |  |  |  |  |  |  |  |  |  |  |  |  |  |  |  |  |  |  |  |  |  |  |  |  |  |  |  |  | *hgo-1* | HomoGentisate Oxidase |
|  |  |  |  |  |  |  |  |  |  |  |  |  |  |  |  |  |  |  |  |  |  |  |  |  |  |  |  |  |  |  |  |  |  |  |  |  |  | *ztf-26* | Zinc finger putative Transcription Factor family |
|  |  |  |  |  |  |  |  |  |  |  |  |  |  |  |  |  |  |  |  |  |  |  |  |  |  |  |  |  |  |  |  |  |  |  |  |  |  | F58H1.5 |  |
|  |  |  |  |  |  |  |  |  |  |  |  |  |  |  |  |  |  |  |  |  |  |  |  |  |  |  |  |  |  |  |  |  |  |  |  |  |  | F32D8.15 |  |
|  |  |  |  |  |  |  |  |  |  |  |  |  |  |  |  |  |  |  |  |  |  |  |  |  |  |  |  |  |  |  |  |  |  |  |  |  |  | F36G3.3 |  |
|  |  |  |  |  |  |  |  |  |  |  |  |  |  |  |  |  |  |  |  |  |  |  |  |  |  |  |  |  |  |  |  |  |  |  |  |  |  | H05G16.3 |  |
|  |  |  |  |  |  |  |  |  |  |  |  |  |  |  |  |  |  |  |  |  |  |  |  |  |  |  |  |  |  |  |  |  |  |  |  |  |  | ZC123.6 |  |
|  |  |  |  |  |  |  |  |  |  |  |  |  |  |  |  |  |  |  |  |  |  |  |  |  |  |  |  |  |  |  |  |  |  |  |  |  |  | *pkd-2* | human PKD2 (Polycystic Kidney Disease) related |
|  |  |  |  |  |  |  |  |  |  |  |  |  |  |  |  |  |  |  |  |  |  |  |  |  |  |  |  |  |  |  |  |  |  |  |  |  |  | *cal-6* | CALmodulin related genes |
|  |  |  |  |  |  |  |  |  |  |  |  |  |  |  |  |  |  |  |  |  |  |  |  |  |  |  |  |  |  |  |  |  |  |  |  |  |  | *snap-29* | SNAP (Soluble NSF Attachment Protein) homolog |
|  |  |  |  |  |  |  |  |  |  |  |  |  |  |  |  |  |  |  |  |  |  |  |  |  |  |  |  |  |  |  |  |  |  |  |  |  |  | F39E9.10 |  |
|  |  |  |  |  |  |  |  |  |  |  |  |  |  |  |  |  |  |  |  |  |  |  |  |  |  |  |  |  |  |  |  |  |  |  |  |  |  | K02E2.6 |  |
|  |  |  |  |  |  |  |  |  |  |  |  |  |  |  |  |  |  |  |  |  |  |  |  |  |  |  |  |  |  |  |  |  |  |  |  |  |  | *saeg-2* | Suppressor of Activated EGL-4 |
|  |  |  |  |  |  |  |  |  |  |  |  |  |  |  |  |  |  |  |  |  |  |  |  |  |  |  |  |  |  |  |  |  |  |  |  |  |  | *mcp-1* | Metabolite Control Phosphorylase |
|  |  |  |  |  |  |  |  |  |  |  |  |  |  |  |  |  |  |  |  |  |  |  |  |  |  |  |  |  |  |  |  |  |  |  |  |  |  | F41E6.17 |  |
|  |  |  |  |  |  |  |  |  |  |  |  |  |  |  |  |  |  |  |  |  |  |  |  |  |  |  |  |  |  |  |  |  |  |  |  |  |  | *twk-23* | TWiK family of potassium channels |
|  |  |  |  |  |  |  |  |  |  |  |  |  |  |  |  |  |  |  |  |  |  |  |  |  |  |  |  |  |  |  |  |  |  |  |  |  |  | C35B8.3 |  |
|  |  |  |  |  |  |  |  |  |  |  |  |  |  |  |  |  |  |  |  |  |  |  |  |  |  |  |  |  |  |  |  |  |  |  |  |  |  | H31G24.2 |  |
|  |  |  |  |  |  |  |  |  |  |  |  |  |  |  |  |  |  |  |  |  |  |  |  |  |  |  |  |  |  |  |  |  |  |  |  |  |  | C18B2.4 |  |
|  |  |  |  |  |  |  |  |  |  |  |  |  |  |  |  |  |  |  |  |  |  |  |  |  |  |  |  |  |  |  |  |  |  |  |  |  |  | C16C2.4 |  |
|  |  |  |  |  |  |  |  |  |  |  |  |  |  |  |  |  |  |  |  |  |  |  |  |  |  |  |  |  |  |  |  |  |  |  |  |  |  | T23B12.6 |  |
|  |  |  |  |  |  |  |  |  |  |  |  |  |  |  |  |  |  |  |  |  |  |  |  |  |  |  |  |  |  |  |  |  |  |  |  |  |  | *fbxa-167* | F-box A protein |
|  |  |  |  |  |  |  |  |  |  |  |  |  |  |  |  |  |  |  |  |  |  |  |  |  |  |  |  |  |  |  |  |  |  |  |  |  |  | W04B5.2 |  |
|  |  |  |  |  |  |  |  |  |  |  |  |  |  |  |  |  |  |  |  |  |  |  |  |  |  |  |  |  |  |  |  |  |  |  |  |  |  | F54B3.2 |  |
|  |  |  |  |  |  |  |  |  |  |  |  |  |  |  |  |  |  |  |  |  |  |  |  |  |  |  |  |  |  |  |  |  |  |  |  |  |  | F54B3.4 |  |
|  |  |  |  |  |  |  |  |  |  |  |  |  |  |  |  |  |  |  |  |  |  |  |  |  |  |  |  |  |  |  |  |  |  |  |  |  |  | *srg-7* | Serpentine Receptor, class G (gamma) |
|  |  |  |  |  |  |  |  |  |  |  |  |  |  |  |  |  |  |  |  |  |  |  |  |  |  |  |  |  |  |  |  |  |  |  |  |  |  | *srr-10* | Serpentine Receptor, class R |
|  |  |  |  |  |  |  |  |  |  |  |  |  |  |  |  |  |  |  |  |  |  |  |  |  |  |  |  |  |  |  |  |  |  |  |  |  |  | *plx-2* | PLeXin |
|  |  |  |  |  |  |  |  |  |  |  |  |  |  |  |  |  |  |  |  |  |  |  |  |  |  |  |  |  |  |  |  |  |  |  |  |  |  | *ubql-1* | UBiQuiLin |
|  |  |  |  |  |  |  |  |  |  |  |  |  |  |  |  |  |  |  |  |  |  |  |  |  |  |  |  |  |  |  |  |  |  |  |  |  |  | T19D7.4 |  |
|  |  |  |  |  |  |  |  |  |  |  |  |  |  |  |  |  |  |  |  |  |  |  |  |  |  |  |  |  |  |  |  |  |  |  |  |  |  | *gopc-1* | GOlgi-associated PDZ and Coiled-coil motif protein |
|  |  |  |  |  |  |  |  |  |  |  |  |  |  |  |  |  |  |  |  |  |  |  |  |  |  |  |  |  |  |  |  |  |  |  |  |  |  | ZK849.1 |  |
|  |  |  |  |  |  |  |  |  |  |  |  |  |  |  |  |  |  |  |  |  |  |  |  |  |  |  |  |  |  |  |  |  |  |  |  |  |  | *glb-10* | GLoBin related |
|  |  |  |  |  |  |  |  |  |  |  |  |  |  |  |  |  |  |  |  |  |  |  |  |  |  |  |  |  |  |  |  |  |  |  |  |  |  | C15H9.2 |  |
|  |  |  |  |  |  |  |  |  |  |  |  |  |  |  |  |  |  |  |  |  |  |  |  |  |  |  |  |  |  |  |  |  |  |  |  |  |  | C49C3.11 |  |
|  |  |  |  |  |  |  |  |  |  |  |  |  |  |  |  |  |  |  |  |  |  |  |  |  |  |  |  |  |  |  |  |  |  |  |  |  |  | *tmbi-4* | TMBI (TransMembrane BAX Inhibitor motif protein) homolog |
|  |  |  |  |  |  |  |  |  |  |  |  |  |  |  |  |  |  |  |  |  |  |  |  |  |  |  |  |  |  |  |  |  |  |  |  |  |  | *cah-3* | Carbonic AnHydrase |
|  |  |  |  |  |  |  |  |  |  |  |  |  |  |  |  |  |  |  |  |  |  |  |  |  |  |  |  |  |  |  |  |  |  |  |  |  |  | C43F9.7 |  |
|  |  |  |  |  |  |  |  |  |  |  |  |  |  |  |  |  |  |  |  |  |  |  |  |  |  |  |  |  |  |  |  |  |  |  |  |  |  | C05C12.6 |  |
|  |  |  |  |  |  |  |  |  |  |  |  |  |  |  |  |  |  |  |  |  |  |  |  |  |  |  |  |  |  |  |  |  |  |  |  |  |  | *drn-1* | Di-Ras/Rig/Noey2 Ras-like protein homolog |
|  |  |  |  |  |  |  |  |  |  |  |  |  |  |  |  |  |  |  |  |  |  |  |  |  |  |  |  |  |  |  |  |  |  |  |  |  |  | *linc-149* | Long Intervening Non-Coding RNA |
|  |  |  |  |  |  |  |  |  |  |  |  |  |  |  |  |  |  |  |  |  |  |  |  |  |  |  |  |  |  |  |  |  |  |  |  |  |  | *dlc-2* | Dynein Light Chain |
|  |  |  |  |  |  |  |  |  |  |  |  |  |  |  |  |  |  |  |  |  |  |  |  |  |  |  |  |  |  |  |  |  |  |  |  |  |  | *glb-33* | GLoBin related |
|  |  |  |  |  |  |  |  |  |  |  |  |  |  |  |  |  |  |  |  |  |  |  |  |  |  |  |  |  |  |  |  |  |  |  |  |  |  | *srt-53* | Serpentine Receptor, class T |
|  |  |  |  |  |  |  |  |  |  |  |  |  |  |  |  |  |  |  |  |  |  |  |  |  |  |  |  |  |  |  |  |  |  |  |  |  |  | Y41D4B.24 |  |
|  |  |  |  |  |  |  |  |  |  |  |  |  |  |  |  |  |  |  |  |  |  |  |  |  |  |  |  |  |  |  |  |  |  |  |  |  |  | C17H12.10 |  |
|  |  |  |  |  |  |  |  |  |  |  |  |  |  |  |  |  |  |  |  |  |  |  |  |  |  |  |  |  |  |  |  |  |  |  |  |  |  | C39F7.1 |  |
|  |  |  |  |  |  |  |  |  |  |  |  |  |  |  |  |  |  |  |  |  |  |  |  |  |  |  |  |  |  |  |  |  |  |  |  |  |  | ZK673.11 |  |
|  |  |  |  |  |  |  |  |  |  |  |  |  |  |  |  |  |  |  |  |  |  |  |  |  |  |  |  |  |  |  |  |  |  |  |  |  |  | ZK673.5 |  |
|  |  |  |  |  |  |  |  |  |  |  |  |  |  |  |  |  |  |  |  |  |  |  |  |  |  |  |  |  |  |  |  |  |  |  |  |  |  | ZK673.13 |  |
|  |  |  |  |  |  |  |  |  |  |  |  |  |  |  |  |  |  |  |  |  |  |  |  |  |  |  |  |  |  |  |  |  |  |  |  |  |  | *nca-2* | Novel Channel type/putative Nematode CAlcium channel |
|  |  |  |  |  |  |  |  |  |  |  |  |  |  |  |  |  |  |  |  |  |  |  |  |  |  |  |  |  |  |  |  |  |  |  |  |  |  | *twk-29* | TWiK family of potassium channels |
|  |  |  |  |  |  |  |  |  |  |  |  |  |  |  |  |  |  |  |  |  |  |  |  |  |  |  |  |  |  |  |  |  |  |  |  |  |  | R173.3 |  |
|  |  |  |  |  |  |  |  |  |  |  |  |  |  |  |  |  |  |  |  |  |  |  |  |  |  |  |  |  |  |  |  |  |  |  |  |  |  | *ten-1* | TENeurin |
|  |  |  |  |  |  |  |  |  |  |  |  |  |  |  |  |  |  |  |  |  |  |  |  |  |  |  |  |  |  |  |  |  |  |  |  |  |  | *klp-11* | Kinesin-Like Protein |
|  |  |  |  |  |  |  |  |  |  |  |  |  |  |  |  |  |  |  |  |  |  |  |  |  |  |  |  |  |  |  |  |  |  |  |  |  |  | *tsp-3* | TetraSPanin family |
|  |  |  |  |  |  |  |  |  |  |  |  |  |  |  |  |  |  |  |  |  |  |  |  |  |  |  |  |  |  |  |  |  |  |  |  |  |  | K10C9.1 |  |
|  |  |  |  |  |  |  |  |  |  |  |  |  |  |  |  |  |  |  |  |  |  |  |  |  |  |  |  |  |  |  |  |  |  |  |  |  |  | *rcn-1* | Regulator of CalciNeurin |
|  |  |  |  |  |  |  |  |  |  |  |  |  |  |  |  |  |  |  |  |  |  |  |  |  |  |  |  |  |  |  |  |  |  |  |  |  |  | C14A11.2 |  |
|  |  |  |  |  |  |  |  |  |  |  |  |  |  |  |  |  |  |  |  |  |  |  |  |  |  |  |  |  |  |  |  |  |  |  |  |  |  | F32B5.2 |  |
|  |  |  |  |  |  |  |  |  |  |  |  |  |  |  |  |  |  |  |  |  |  |  |  |  |  |  |  |  |  |  |  |  |  |  |  |  |  | *iglr-1* | IG(immunoglobulin) and LRR(Leucine Rich Repeat) domains |
|  |  |  |  |  |  |  |  |  |  |  |  |  |  |  |  |  |  |  |  |  |  |  |  |  |  |  |  |  |  |  |  |  |  |  |  |  |  | B0207.2 |  |
|  |  |  |  |  |  |  |  |  |  |  |  |  |  |  |  |  |  |  |  |  |  |  |  |  |  |  |  |  |  |  |  |  |  |  |  |  |  | F46B6.2 |  |
|  |  |  |  |  |  |  |  |  |  |  |  |  |  |  |  |  |  |  |  |  |  |  |  |  |  |  |  |  |  |  |  |  |  |  |  |  |  | *sol-1* | Suppressor Of Lurcher movement defect |
|  |  |  |  |  |  |  |  |  |  |  |  |  |  |  |  |  |  |  |  |  |  |  |  |  |  |  |  |  |  |  |  |  |  |  |  |  |  | Y17D7C.3 |  |
|  |  |  |  |  |  |  |  |  |  |  |  |  |  |  |  |  |  |  |  |  |  |  |  |  |  |  |  |  |  |  |  |  |  |  |  |  |  | *rskd-1* | Ribosomal protein S6 Kinase Delta homolog |
|  |  |  |  |  |  |  |  |  |  |  |  |  |  |  |  |  |  |  |  |  |  |  |  |  |  |  |  |  |  |  |  |  |  |  |  |  |  | F30A10.3 |  |
|  |  |  |  |  |  |  |  |  |  |  |  |  |  |  |  |  |  |  |  |  |  |  |  |  |  |  |  |  |  |  |  |  |  |  |  |  |  | Y32H12A.7 |  |
|  |  |  |  |  |  |  |  |  |  |  |  |  |  |  |  |  |  |  |  |  |  |  |  |  |  |  |  |  |  |  |  |  |  |  |  |  |  | *rilp-1* | RILP (Rab7-Interacting Lysosomal Protein) homolog |
|  |  |  |  |  |  |  |  |  |  |  |  |  |  |  |  |  |  |  |  |  |  |  |  |  |  |  |  |  |  |  |  |  |  |  |  |  |  | Y34B4A.8 |  |
|  |  |  |  |  |  |  |  |  |  |  |  |  |  |  |  |  |  |  |  |  |  |  |  |  |  |  |  |  |  |  |  |  |  |  |  |  |  | K11C4.2 |  |
|  |  |  |  |  |  |  |  |  |  |  |  |  |  |  |  |  |  |  |  |  |  |  |  |  |  |  |  |  |  |  |  |  |  |  |  |  |  | R09F10.5 |  |
|  |  |  |  |  |  |  |  |  |  |  |  |  |  |  |  |  |  |  |  |  |  |  |  |  |  |  |  |  |  |  |  |  |  |  |  |  |  | K07A9.3 |  |
|  |  |  |  |  |  |  |  |  |  |  |  |  |  |  |  |  |  |  |  |  |  |  |  |  |  |  |  |  |  |  |  |  |  |  |  |  |  | C24B5.1 |  |
|  |  |  |  |  |  |  |  |  |  |  |  |  |  |  |  |  |  |  |  |  |  |  |  |  |  |  |  |  |  |  |  |  |  |  |  |  |  | *nlg-1* | NeuroLiGin family |
|  |  |  |  |  |  |  |  |  |  |  |  |  |  |  |  |  |  |  |  |  |  |  |  |  |  |  |  |  |  |  |  |  |  |  |  |  |  | Y71H2AM.10 |  |
|  |  |  |  |  |  |  |  |  |  |  |  |  |  |  |  |  |  |  |  |  |  |  |  |  |  |  |  |  |  |  |  |  |  |  |  |  |  | *unc-9* | UNCoordinated |
|  |  |  |  |  |  |  |  |  |  |  |  |  |  |  |  |  |  |  |  |  |  |  |  |  |  |  |  |  |  |  |  |  |  |  |  |  |  | F52D10.6 |  |
|  |  |  |  |  |  |  |  |  |  |  |  |  |  |  |  |  |  |  |  |  |  |  |  |  |  |  |  |  |  |  |  |  |  |  |  |  |  | F25E5.16 |  |
|  |  |  |  |  |  |  |  |  |  |  |  |  |  |  |  |  |  |  |  |  |  |  |  |  |  |  |  |  |  |  |  |  |  |  |  |  |  | T04A8.18 |  |
|  |  |  |  |  |  |  |  |  |  |  |  |  |  |  |  |  |  |  |  |  |  |  |  |  |  |  |  |  |  |  |  |  |  |  |  |  |  | *frm-3* | FERM domain (protein4.1-ezrin-radixin-moesin) family |
|  |  |  |  |  |  |  |  |  |  |  |  |  |  |  |  |  |  |  |  |  |  |  |  |  |  |  |  |  |  |  |  |  |  |  |  |  |  | *shw-3* | SHaW family of potassium channels |
|  |  |  |  |  |  |  |  |  |  |  |  |  |  |  |  |  |  |  |  |  |  |  |  |  |  |  |  |  |  |  |  |  |  |  |  |  |  | *glr-1* | GLutamate Receptor family (AMPA) |
|  |  |  |  |  |  |  |  |  |  |  |  |  |  |  |  |  |  |  |  |  |  |  |  |  |  |  |  |  |  |  |  |  |  |  |  |  |  | *lron-13* | eLRR (extracellular Leucine-Rich Repeat) ONly |
|  |  |  |  |  |  |  |  |  |  |  |  |  |  |  |  |  |  |  |  |  |  |  |  |  |  |  |  |  |  |  |  |  |  |  |  |  |  | *gar-2* | G-protein-linked Acetylcholine Receptor |
|  |  |  |  |  |  |  |  |  |  |  |  |  |  |  |  |  |  |  |  |  |  |  |  |  |  |  |  |  |  |  |  |  |  |  |  |  |  | *cah-6* | Carbonic AnHydrase |
|  |  |  |  |  |  |  |  |  |  |  |  |  |  |  |  |  |  |  |  |  |  |  |  |  |  |  |  |  |  |  |  |  |  |  |  |  |  | W05H12.1 |  |
|  |  |  |  |  |  |  |  |  |  |  |  |  |  |  |  |  |  |  |  |  |  |  |  |  |  |  |  |  |  |  |  |  |  |  |  |  |  | *mpz-1* | Multiple PDZ domain protein |
|  |  |  |  |  |  |  |  |  |  |  |  |  |  |  |  |  |  |  |  |  |  |  |  |  |  |  |  |  |  |  |  |  |  |  |  |  |  | *dhs-17* | DeHydrogenases, Short chain |
|  |  |  |  |  |  |  |  |  |  |  |  |  |  |  |  |  |  |  |  |  |  |  |  |  |  |  |  |  |  |  |  |  |  |  |  |  |  | *sto-4* | STOmatin |
|  |  |  |  |  |  |  |  |  |  |  |  |  |  |  |  |  |  |  |  |  |  |  |  |  |  |  |  |  |  |  |  |  |  |  |  |  |  | T14B4.8 |  |
|  |  |  |  |  |  |  |  |  |  |  |  |  |  |  |  |  |  |  |  |  |  |  |  |  |  |  |  |  |  |  |  |  |  |  |  |  |  | *ncs-2* | Neuronal Calcium Sensor family |
|  |  |  |  |  |  |  |  |  |  |  |  |  |  |  |  |  |  |  |  |  |  |  |  |  |  |  |  |  |  |  |  |  |  |  |  |  |  | *nlf-1* | NCA Localization Factor |
|  |  |  |  |  |  |  |  |  |  |  |  |  |  |  |  |  |  |  |  |  |  |  |  |  |  |  |  |  |  |  |  |  |  |  |  |  |  | *unc-57* | UNCoordinated |
|  |  |  |  |  |  |  |  |  |  |  |  |  |  |  |  |  |  |  |  |  |  |  |  |  |  |  |  |  |  |  |  |  |  |  |  |  |  | *lin-2* | abnormal cell LINeage |
|  |  |  |  |  |  |  |  |  |  |  |  |  |  |  |  |  |  |  |  |  |  |  |  |  |  |  |  |  |  |  |  |  |  |  |  |  |  | *aex-6* | ABoc, EXpulsion (defecation) defective |
|  |  |  |  |  |  |  |  |  |  |  |  |  |  |  |  |  |  |  |  |  |  |  |  |  |  |  |  |  |  |  |  |  |  |  |  |  |  | *snn-1* | SyNapsiN |
|  |  |  |  |  |  |  |  |  |  |  |  |  |  |  |  |  |  |  |  |  |  |  |  |  |  |  |  |  |  |  |  |  |  |  |  |  |  | *rab-3* | RAB family |
|  |  |  |  |  |  |  |  |  |  |  |  |  |  |  |  |  |  |  |  |  |  |  |  |  |  |  |  |  |  |  |  |  |  |  |  |  |  | T14G11.1 |  |
|  |  |  |  |  |  |  |  |  |  |  |  |  |  |  |  |  |  |  |  |  |  |  |  |  |  |  |  |  |  |  |  |  |  |  |  |  |  | F53C3.3 |  |
|  |  |  |  |  |  |  |  |  |  |  |  |  |  |  |  |  |  |  |  |  |  |  |  |  |  |  |  |  |  |  |  |  |  |  |  |  |  | *ric-4* | Resistance to Inhibitors of Cholinesterase |
|  |  |  |  |  |  |  |  |  |  |  |  |  |  |  |  |  |  |  |  |  |  |  |  |  |  |  |  |  |  |  |  |  |  |  |  |  |  | F46C5.4 |  |
|  |  |  |  |  |  |  |  |  |  |  |  |  |  |  |  |  |  |  |  |  |  |  |  |  |  |  |  |  |  |  |  |  |  |  |  |  |  | F46C5.7 |  |
|  |  |  |  |  |  |  |  |  |  |  |  |  |  |  |  |  |  |  |  |  |  |  |  |  |  |  |  |  |  |  |  |  |  |  |  |  |  | Y47G6A.7 |  |
|  |  |  |  |  |  |  |  |  |  |  |  |  |  |  |  |  |  |  |  |  |  |  |  |  |  |  |  |  |  |  |  |  |  |  |  |  |  | *srv-24* | Serpentine Receptor, class V |
|  |  |  |  |  |  |  |  |  |  |  |  |  |  |  |  |  |  |  |  |  |  |  |  |  |  |  |  |  |  |  |  |  |  |  |  |  |  | *aptf-4* | AP-2 Transcription Factor family |
|  |  |  |  |  |  |  |  |  |  |  |  |  |  |  |  |  |  |  |  |  |  |  |  |  |  |  |  |  |  |  |  |  |  |  |  |  |  | *nsf-1* | NSF (N-ethylmaleimide sensitive secretion factor) homolog |
|  |  |  |  |  |  |  |  |  |  |  |  |  |  |  |  |  |  |  |  |  |  |  |  |  |  |  |  |  |  |  |  |  |  |  |  |  |  | *klp-4* | Kinesin-Like Protein |
|  |  |  |  |  |  |  |  |  |  |  |  |  |  |  |  |  |  |  |  |  |  |  |  |  |  |  |  |  |  |  |  |  |  |  |  |  |  | *sulp-1* | SULfate Permease family |
|  |  |  |  |  |  |  |  |  |  |  |  |  |  |  |  |  |  |  |  |  |  |  |  |  |  |  |  |  |  |  |  |  |  |  |  |  |  | ZK54.3 |  |
|  |  |  |  |  |  |  |  |  |  |  |  |  |  |  |  |  |  |  |  |  |  |  |  |  |  |  |  |  |  |  |  |  |  |  |  |  |  | *inx-1* | INneXin |
|  |  |  |  |  |  |  |  |  |  |  |  |  |  |  |  |  |  |  |  |  |  |  |  |  |  |  |  |  |  |  |  |  |  |  |  |  |  | *sto-2* | STOmatin |
|  |  |  |  |  |  |  |  |  |  |  |  |  |  |  |  |  |  |  |  |  |  |  |  |  |  |  |  |  |  |  |  |  |  |  |  |  |  | F07H5.7 |  |
|  |  |  |  |  |  |  |  |  |  |  |  |  |  |  |  |  |  |  |  |  |  |  |  |  |  |  |  |  |  |  |  |  |  |  |  |  |  | *avr-14* | altered AVeRmectin sensitivity |
|  |  |  |  |  |  |  |  |  |  |  |  |  |  |  |  |  |  |  |  |  |  |  |  |  |  |  |  |  |  |  |  |  |  |  |  |  |  | *rpy-1* | RaPsYn |
|  |  |  |  |  |  |  |  |  |  |  |  |  |  |  |  |  |  |  |  |  |  |  |  |  |  |  |  |  |  |  |  |  |  |  |  |  |  | T23D8.3 |  |
|  |  |  |  |  |  |  |  |  |  |  |  |  |  |  |  |  |  |  |  |  |  |  |  |  |  |  |  |  |  |  |  |  |  |  |  |  |  | *tsp-7* | TetraSPanin family |
|  |  |  |  |  |  |  |  |  |  |  |  |  |  |  |  |  |  |  |  |  |  |  |  |  |  |  |  |  |  |  |  |  |  |  |  |  |  | F53C3.6 |  |
|  |  |  |  |  |  |  |  |  |  |  |  |  |  |  |  |  |  |  |  |  |  |  |  |  |  |  |  |  |  |  |  |  |  |  |  |  |  | *unc-8* | UNCoordinated |
|  |  |  |  |  |  |  |  |  |  |  |  |  |  |  |  |  |  |  |  |  |  |  |  |  |  |  |  |  |  |  |  |  |  |  |  |  |  | *hum-5* | Heavy chain, Unconventional Myosin |
|  |  |  |  |  |  |  |  |  |  |  |  |  |  |  |  |  |  |  |  |  |  |  |  |  |  |  |  |  |  |  |  |  |  |  |  |  |  | *acy-1* | Adenylyl CYclase |
|  |  |  |  |  |  |  |  |  |  |  |  |  |  |  |  |  |  |  |  |  |  |  |  |  |  |  |  |  |  |  |  |  |  |  |  |  |  | *unc-80* | UNCoordinated |
|  |  |  |  |  |  |  |  |  |  |  |  |  |  |  |  |  |  |  |  |  |  |  |  |  |  |  |  |  |  |  |  |  |  |  |  |  |  | *rab-14* | RAB family |
|  |  |  |  |  |  |  |  |  |  |  |  |  |  |  |  |  |  |  |  |  |  |  |  |  |  |  |  |  |  |  |  |  |  |  |  |  |  | F32D1.3 |  |
|  |  |  |  |  |  |  |  |  |  |  |  |  |  |  |  |  |  |  |  |  |  |  |  |  |  |  |  |  |  |  |  |  |  |  |  |  |  | *apa-2* | AdaPtin, Alpha chain (clathrin associated complex) |
|  |  |  |  |  |  |  |  |  |  |  |  |  |  |  |  |  |  |  |  |  |  |  |  |  |  |  |  |  |  |  |  |  |  |  |  |  |  | C02F5.12 |  |
|  |  |  |  |  |  |  |  |  |  |  |  |  |  |  |  |  |  |  |  |  |  |  |  |  |  |  |  |  |  |  |  |  |  |  |  |  |  | *ric-3* | Resistance to Inhibitors of Cholinesterase |
|  |  |  |  |  |  |  |  |  |  |  |  |  |  |  |  |  |  |  |  |  |  |  |  |  |  |  |  |  |  |  |  |  |  |  |  |  |  | Y67A10A.7 |  |
|  |  |  |  |  |  |  |  |  |  |  |  |  |  |  |  |  |  |  |  |  |  |  |  |  |  |  |  |  |  |  |  |  |  |  |  |  |  | *mkk-4* | MKK (MAP Kinase Kinase) homolog |
|  |  |  |  |  |  |  |  |  |  |  |  |  |  |  |  |  |  |  |  |  |  |  |  |  |  |  |  |  |  |  |  |  |  |  |  |  |  | C26D10.6 |  |
|  |  |  |  |  |  |  |  |  |  |  |  |  |  |  |  |  |  |  |  |  |  |  |  |  |  |  |  |  |  |  |  |  |  |  |  |  |  | *tag-257* | Temporarily Assigned Gene name |
|  |  |  |  |  |  |  |  |  |  |  |  |  |  |  |  |  |  |  |  |  |  |  |  |  |  |  |  |  |  |  |  |  |  |  |  |  |  | K02E10.7 |  |
|  |  |  |  |  |  |  |  |  |  |  |  |  |  |  |  |  |  |  |  |  |  |  |  |  |  |  |  |  |  |  |  |  |  |  |  |  |  | *ceh-58* | C. Elegans Homeobox |
|  |  |  |  |  |  |  |  |  |  |  |  |  |  |  |  |  |  |  |  |  |  |  |  |  |  |  |  |  |  |  |  |  |  |  |  |  |  | *daf-19* | abnormal DAuer Formation |
|  |  |  |  |  |  |  |  |  |  |  |  |  |  |  |  |  |  |  |  |  |  |  |  |  |  |  |  |  |  |  |  |  |  |  |  |  |  | C14A11.5 |  |
|  |  |  |  |  |  |  |  |  |  |  |  |  |  |  |  |  |  |  |  |  |  |  |  |  |  |  |  |  |  |  |  |  |  |  |  |  |  | F32D8.12 |  |
|  |  |  |  |  |  |  |  |  |  |  |  |  |  |  |  |  |  |  |  |  |  |  |  |  |  |  |  |  |  |  |  |  |  |  |  |  |  | C35E7.4 |  |
|  |  |  |  |  |  |  |  |  |  |  |  |  |  |  |  |  |  |  |  |  |  |  |  |  |  |  |  |  |  |  |  |  |  |  |  |  |  | *acc-4* | Acetylcholine-gated Chloride Channel |
|  |  |  |  |  |  |  |  |  |  |  |  |  |  |  |  |  |  |  |  |  |  |  |  |  |  |  |  |  |  |  |  |  |  |  |  |  |  | C01G6.4 |  |
|  |  |  |  |  |  |  |  |  |  |  |  |  |  |  |  |  |  |  |  |  |  |  |  |  |  |  |  |  |  |  |  |  |  |  |  |  |  | C25D7.10 |  |
|  |  |  |  |  |  |  |  |  |  |  |  |  |  |  |  |  |  |  |  |  |  |  |  |  |  |  |  |  |  |  |  |  |  |  |  |  |  | *anat-1* | AANAT (Arylalkylamine N-AcetylTransferase) homolog |
|  |  |  |  |  |  |  |  |  |  |  |  |  |  |  |  |  |  |  |  |  |  |  |  |  |  |  |  |  |  |  |  |  |  |  |  |  |  | Y54E10BL.3 |  |
|  |  |  |  |  |  |  |  |  |  |  |  |  |  |  |  |  |  |  |  |  |  |  |  |  |  |  |  |  |  |  |  |  |  |  |  |  |  | *unc-108* | UNCoordinated |
|  |  |  |  |  |  |  |  |  |  |  |  |  |  |  |  |  |  |  |  |  |  |  |  |  |  |  |  |  |  |  |  |  |  |  |  |  |  | C53D5.3 |  |
|  |  |  |  |  |  |  |  |  |  |  |  |  |  |  |  |  |  |  |  |  |  |  |  |  |  |  |  |  |  |  |  |  |  |  |  |  |  | T12B3.4 |  |
|  |  |  |  |  |  |  |  |  |  |  |  |  |  |  |  |  |  |  |  |  |  |  |  |  |  |  |  |  |  |  |  |  |  |  |  |  |  | Y60A3A.8 |  |
|  |  |  |  |  |  |  |  |  |  |  |  |  |  |  |  |  |  |  |  |  |  |  |  |  |  |  |  |  |  |  |  |  |  |  |  |  |  | Y87G2A.11 |  |
|  |  |  |  |  |  |  |  |  |  |  |  |  |  |  |  |  |  |  |  |  |  |  |  |  |  |  |  |  |  |  |  |  |  |  |  |  |  | *zer-1* | Zyg Eleven Related |
|  |  |  |  |  |  |  |  |  |  |  |  |  |  |  |  |  |  |  |  |  |  |  |  |  |  |  |  |  |  |  |  |  |  |  |  |  |  | *hda-4* | Histone DeAcetylase |
|  |  |  |  |  |  |  |  |  |  |  |  |  |  |  |  |  |  |  |  |  |  |  |  |  |  |  |  |  |  |  |  |  |  |  |  |  |  | *gpi-1* | Glucose-6-Phosphate Isomerase |
|  |  |  |  |  |  |  |  |  |  |  |  |  |  |  |  |  |  |  |  |  |  |  |  |  |  |  |  |  |  |  |  |  |  |  |  |  |  | *abts-1* | Anion/Bicarbonate TranSporter family |
|  |  |  |  |  |  |  |  |  |  |  |  |  |  |  |  |  |  |  |  |  |  |  |  |  |  |  |  |  |  |  |  |  |  |  |  |  |  | F15A8.6 |  |
|  |  |  |  |  |  |  |  |  |  |  |  |  |  |  |  |  |  |  |  |  |  |  |  |  |  |  |  |  |  |  |  |  |  |  |  |  |  | ZC581.9 |  |
|  |  |  |  |  |  |  |  |  |  |  |  |  |  |  |  |  |  |  |  |  |  |  |  |  |  |  |  |  |  |  |  |  |  |  |  |  |  | *eat-6* | EATing: abnormal pharyngeal pumping |
|  |  |  |  |  |  |  |  |  |  |  |  |  |  |  |  |  |  |  |  |  |  |  |  |  |  |  |  |  |  |  |  |  |  |  |  |  |  | *tom-1* | TOMosyn synaptic protein |
|  |  |  |  |  |  |  |  |  |  |  |  |  |  |  |  |  |  |  |  |  |  |  |  |  |  |  |  |  |  |  |  |  |  |  |  |  |  | F35C8.9 |  |
|  |  |  |  |  |  |  |  |  |  |  |  |  |  |  |  |  |  |  |  |  |  |  |  |  |  |  |  |  |  |  |  |  |  |  |  |  |  | *kin-4* | protein KINase |
|  |  |  |  |  |  |  |  |  |  |  |  |  |  |  |  |  |  |  |  |  |  |  |  |  |  |  |  |  |  |  |  |  |  |  |  |  |  | Y48C3A.5 |  |
|  |  |  |  |  |  |  |  |  |  |  |  |  |  |  |  |  |  |  |  |  |  |  |  |  |  |  |  |  |  |  |  |  |  |  |  |  |  | C11D2.3 |  |
|  |  |  |  |  |  |  |  |  |  |  |  |  |  |  |  |  |  |  |  |  |  |  |  |  |  |  |  |  |  |  |  |  |  |  |  |  |  | C06H5.8 |  |
|  |  |  |  |  |  |  |  |  |  |  |  |  |  |  |  |  |  |  |  |  |  |  |  |  |  |  |  |  |  |  |  |  |  |  |  |  |  | T06F4.1 |  |
|  |  |  |  |  |  |  |  |  |  |  |  |  |  |  |  |  |  |  |  |  |  |  |  |  |  |  |  |  |  |  |  |  |  |  |  |  |  | *lim-9* | LIM domain family |
|  |  |  |  |  |  |  |  |  |  |  |  |  |  |  |  |  |  |  |  |  |  |  |  |  |  |  |  |  |  |  |  |  |  |  |  |  |  | *unc-1* | UNCoordinated |
|  |  |  |  |  |  |  |  |  |  |  |  |  |  |  |  |  |  |  |  |  |  |  |  |  |  |  |  |  |  |  |  |  |  |  |  |  |  | *kin-2* | protein KINase |
|  |  |  |  |  |  |  |  |  |  |  |  |  |  |  |  |  |  |  |  |  |  |  |  |  |  |  |  |  |  |  |  |  |  |  |  |  |  | *cdka-1* | Cyclin-Dependent Kinase 5 Activating protein homolog |
|  |  |  |  |  |  |  |  |  |  |  |  |  |  |  |  |  |  |  |  |  |  |  |  |  |  |  |  |  |  |  |  |  |  |  |  |  |  | *sng-1* | SyNaptoGyrin family |
|  |  |  |  |  |  |  |  |  |  |  |  |  |  |  |  |  |  |  |  |  |  |  |  |  |  |  |  |  |  |  |  |  |  |  |  |  |  | R04E5.7 |  |
|  |  |  |  |  |  |  |  |  |  |  |  |  |  |  |  |  |  |  |  |  |  |  |  |  |  |  |  |  |  |  |  |  |  |  |  |  |  | R07A4.2 |  |
|  |  |  |  |  |  |  |  |  |  |  |  |  |  |  |  |  |  |  |  |  |  |  |  |  |  |  |  |  |  |  |  |  |  |  |  |  |  | C31H5.5 |  |
|  |  |  |  |  |  |  |  |  |  |  |  |  |  |  |  |  |  |  |  |  |  |  |  |  |  |  |  |  |  |  |  |  |  |  |  |  |  | *nrx-1* | NeuReXin related |
|  |  |  |  |  |  |  |  |  |  |  |  |  |  |  |  |  |  |  |  |  |  |  |  |  |  |  |  |  |  |  |  |  |  |  |  |  |  | C29A12.7 |  |
|  |  |  |  |  |  |  |  |  |  |  |  |  |  |  |  |  |  |  |  |  |  |  |  |  |  |  |  |  |  |  |  |  |  |  |  |  |  | *glb-7* | GLoBin related |
|  |  |  |  |  |  |  |  |  |  |  |  |  |  |  |  |  |  |  |  |  |  |  |  |  |  |  |  |  |  |  |  |  |  |  |  |  |  | K03B4.4 |  |
|  |  |  |  |  |  |  |  |  |  |  |  |  |  |  |  |  |  |  |  |  |  |  |  |  |  |  |  |  |  |  |  |  |  |  |  |  |  | *glb-13* | GLoBin related |
|  |  |  |  |  |  |  |  |  |  |  |  |  |  |  |  |  |  |  |  |  |  |  |  |  |  |  |  |  |  |  |  |  |  |  |  |  |  | F19H6.7 |  |
|  |  |  |  |  |  |  |  |  |  |  |  |  |  |  |  |  |  |  |  |  |  |  |  |  |  |  |  |  |  |  |  |  |  |  |  |  |  | *gcy-28* | Guanylyl CYclase |
|  |  |  |  |  |  |  |  |  |  |  |  |  |  |  |  |  |  |  |  |  |  |  |  |  |  |  |  |  |  |  |  |  |  |  |  |  |  | C29A12.17 |  |
|  |  |  |  |  |  |  |  |  |  |  |  |  |  |  |  |  |  |  |  |  |  |  |  |  |  |  |  |  |  |  |  |  |  |  |  |  |  | Y41E3.7 |  |
|  |  |  |  |  |  |  |  |  |  |  |  |  |  |  |  |  |  |  |  |  |  |  |  |  |  |  |  |  |  |  |  |  |  |  |  |  |  | Y71H10B.1 |  |
|  |  |  |  |  |  |  |  |  |  |  |  |  |  |  |  |  |  |  |  |  |  |  |  |  |  |  |  |  |  |  |  |  |  |  |  |  |  | *olrn-1* | Olfactory LeaRNing defective |
|  |  |  |  |  |  |  |  |  |  |  |  |  |  |  |  |  |  |  |  |  |  |  |  |  |  |  |  |  |  |  |  |  |  |  |  |  |  | C03H5.6 |  |
|  |  |  |  |  |  |  |  |  |  |  |  |  |  |  |  |  |  |  |  |  |  |  |  |  |  |  |  |  |  |  |  |  |  |  |  |  |  | *cha-1* | abnormal CHoline Acetyltransferase |
|  |  |  |  |  |  |  |  |  |  |  |  |  |  |  |  |  |  |  |  |  |  |  |  |  |  |  |  |  |  |  |  |  |  |  |  |  |  | *snt-1* | SyNapTotagmin |
|  |  |  |  |  |  |  |  |  |  |  |  |  |  |  |  |  |  |  |  |  |  |  |  |  |  |  |  |  |  |  |  |  |  |  |  |  |  | *fbxc-41* | F-box C protein |
|  |  |  |  |  |  |  |  |  |  |  |  |  |  |  |  |  |  |  |  |  |  |  |  |  |  |  |  |  |  |  |  |  |  |  |  |  |  | T27C4.1 |  |
|  |  |  |  |  |  |  |  |  |  |  |  |  |  |  |  |  |  |  |  |  |  |  |  |  |  |  |  |  |  |  |  |  |  |  |  |  |  | *frm-8* | FERM domain (protein4.1-ezrin-radixin-moesin) family |
|  |  |  |  |  |  |  |  |  |  |  |  |  |  |  |  |  |  |  |  |  |  |  |  |  |  |  |  |  |  |  |  |  |  |  |  |  |  | T12A7.2 |  |
|  |  |  |  |  |  |  |  |  |  |  |  |  |  |  |  |  |  |  |  |  |  |  |  |  |  |  |  |  |  |  |  |  |  |  |  |  |  | *snt-4* | SyNapTotagmin |
|  |  |  |  |  |  |  |  |  |  |  |  |  |  |  |  |  |  |  |  |  |  |  |  |  |  |  |  |  |  |  |  |  |  |  |  |  |  | *nlp-14* | Neuropeptide-Like Protein |
|  |  |  |  |  |  |  |  |  |  |  |  |  |  |  |  |  |  |  |  |  |  |  |  |  |  |  |  |  |  |  |  |  |  |  |  |  |  | F59D12.1 |  |
|  |  |  |  |  |  |  |  |  |  |  |  |  |  |  |  |  |  |  |  |  |  |  |  |  |  |  |  |  |  |  |  |  |  |  |  |  |  | *qui-1* | QUInine non-avoider |
|  |  |  |  |  |  |  |  |  |  |  |  |  |  |  |  |  |  |  |  |  |  |  |  |  |  |  |  |  |  |  |  |  |  |  |  |  |  | *tag-180* | Temporarily Assigned Gene name |
|  |  |  |  |  |  |  |  |  |  |  |  |  |  |  |  |  |  |  |  |  |  |  |  |  |  |  |  |  |  |  |  |  |  |  |  |  |  | ZK54.1 |  |
|  |  |  |  |  |  |  |  |  |  |  |  |  |  |  |  |  |  |  |  |  |  |  |  |  |  |  |  |  |  |  |  |  |  |  |  |  |  | *tag-168* | Temporarily Assigned Gene name |
|  |  |  |  |  |  |  |  |  |  |  |  |  |  |  |  |  |  |  |  |  |  |  |  |  |  |  |  |  |  |  |  |  |  |  |  |  |  | *unc-18* | UNCoordinated |
|  |  |  |  |  |  |  |  |  |  |  |  |  |  |  |  |  |  |  |  |  |  |  |  |  |  |  |  |  |  |  |  |  |  |  |  |  |  | *rgs-6* | Regulator of G protein Signaling |
|  |  |  |  |  |  |  |  |  |  |  |  |  |  |  |  |  |  |  |  |  |  |  |  |  |  |  |  |  |  |  |  |  |  |  |  |  |  | *ckr-1* | CholecystoKinin Receptor homolog |
|  |  |  |  |  |  |  |  |  |  |  |  |  |  |  |  |  |  |  |  |  |  |  |  |  |  |  |  |  |  |  |  |  |  |  |  |  |  | *daf-3* | abnormal DAuer Formation |
|  |  |  |  |  |  |  |  |  |  |  |  |  |  |  |  |  |  |  |  |  |  |  |  |  |  |  |  |  |  |  |  |  |  |  |  |  |  | *sol-2* | Suppressor Of Lurcher movement defect |
|  |  |  |  |  |  |  |  |  |  |  |  |  |  |  |  |  |  |  |  |  |  |  |  |  |  |  |  |  |  |  |  |  |  |  |  |  |  | *unc-10* | UNCoordinated |
|  |  |  |  |  |  |  |  |  |  |  |  |  |  |  |  |  |  |  |  |  |  |  |  |  |  |  |  |  |  |  |  |  |  |  |  |  |  | *marc-5* | MARCH (Membrane-Associated Ring finger (C3HC4)) homolog |
|  |  |  |  |  |  |  |  |  |  |  |  |  |  |  |  |  |  |  |  |  |  |  |  |  |  |  |  |  |  |  |  |  |  |  |  |  |  | F25E5.7 |  |
|  |  |  |  |  |  |  |  |  |  |  |  |  |  |  |  |  |  |  |  |  |  |  |  |  |  |  |  |  |  |  |  |  |  |  |  |  |  | *pct-1* | PCTAIRE class cell cycle kinase |
|  |  |  |  |  |  |  |  |  |  |  |  |  |  |  |  |  |  |  |  |  |  |  |  |  |  |  |  |  |  |  |  |  |  |  |  |  |  | C35C5.10 |  |
|  |  |  |  |  |  |  |  |  |  |  |  |  |  |  |  |  |  |  |  |  |  |  |  |  |  |  |  |  |  |  |  |  |  |  |  |  |  | *glr-5* | GLutamate Receptor family (AMPA) |
|  |  |  |  |  |  |  |  |  |  |  |  |  |  |  |  |  |  |  |  |  |  |  |  |  |  |  |  |  |  |  |  |  |  |  |  |  |  | *acc-1* | Acetylcholine-gated Chloride Channel |
|  |  |  |  |  |  |  |  |  |  |  |  |  |  |  |  |  |  |  |  |  |  |  |  |  |  |  |  |  |  |  |  |  |  |  |  |  |  | *lgc-47* | Ligand-Gated ion Channel |
|  |  |  |  |  |  |  |  |  |  |  |  |  |  |  |  |  |  |  |  |  |  |  |  |  |  |  |  |  |  |  |  |  |  |  |  |  |  | F09B9.5 |  |

### Phenotypes enriched

|  |  |  |  |
| --- | --- | --- | --- |
| **Group name** | **Number in cluster** | **Enrichment** | **FDR corrected p** |
| acetylcholinesterase inhibitor response variant | 21 | 9.15 | 6.29e-11 |
| organism environmental stimulus response variant | 48 | 3.52 | 1.71e-10 |
| aldicarb response variant | 19 | 9.02 | 1.23e-09 |
| locomotion variant | 36 | 4.19 | 1.95e-09 |
| movement variant | 36 | 4.07 | 4.48e-09 |
| chemical response variant | 36 | 4.04 | 5.56e-09 |
| drug response variant | 30 | 4.79 | 6.09e-09 |
| drug resistant | 23 | 5.65 | 7.89e-08 |
| locomotor coordination variant | 23 | 5.65 | 7.89e-08 |
| behavior variant | 52 | 2.74 | 1.32e-07 |
| organism behavior variant | 52 | 2.74 | 1.32e-07 |
| acetylcholinesterase inhibitor resistant | 13 | 11.69 | 1.62e-07 |
| aldicarb resistant | 11 | 11.34 | 5.98e-06 |
| aldicarb response variant (RNAi) | 23 | 4.40 | 8.40e-06 |
| acetylcholinesterase inhibitor response variant (RNAi) | 23 | 4.40 | 8.40e-06 |
| body posture variant | 13 | 8.33 | 1.16e-05 |
| pharyngeal pumping reduced | 10 | 11.42 | 2.74e-05 |
| activity level variant | 19 | 4.90 | 3.30e-05 |
| drug hypersensitive | 14 | 6.73 | 4.71e-05 |
| drug resistant (RNAi) | 23 | 3.94 | 6.07e-05 |
| chemical response variant (RNAi) | 27 | 3.38 | 9.09e-05 |
| aldicarb resistant (RNAi) | 21 | 4.15 | 9.64e-05 |
| acetylcholinesterase inhibitor resistant (RNAi) | 21 | 4.15 | 9.64e-05 |
| drug response variant (RNAi) | 26 | 3.43 | 1.15e-04 |
| pharyngeal pumping irregular | 7 | 17.41 | 1.51e-04 |
| organism homeostasis metabolism variant | 54 | 2.11 | 2.98e-04 |
| pharyngeal pumping variant | 12 | 6.26 | 8.18e-04 |
| sluggish | 13 | 5.61 | 9.81e-04 |
| organism physiology variant | 54 | 2.01 | 1.10e-03 |
| synapse morphology variant | 8 | 10.25 | 1.37e-03 |
| pharyngeal pumping rate variant | 10 | 7.05 | 2.16e-03 |
| locomotion reduced | 14 | 4.52 | 4.21e-03 |
| thin | 8 | 8.67 | 4.56e-03 |
| coiler | 8 | 8.67 | 4.56e-03 |
| associative learning variant | 4 | 21.14 | 2.33e-02 |

### Anatomy terms enriched

|  |  |  |  |
| --- | --- | --- | --- |
| **Group name** | **Number in cluster** | **Enrichment** | **FDR corrected p** |
| somatic nervous system | 88 | 3.10 | 1.24e-17 |
| ventral cord neuron | 55 | 3.87 | 1.66e-13 |
| somatic neuron | 65 | 3.33 | 2.39e-13 |
| neuron | 121 | 2.17 | 3.05e-13 |
| tail ganglion | 78 | 2.86 | 3.65e-13 |
| nervous system | 113 | 2.24 | 3.83e-13 |
| tail neuron | 77 | 2.87 | 4.89e-13 |
| ganglion | 87 | 2.57 | 1.78e-12 |
| nerve ring | 51 | 3.72 | 7.85e-12 |
| tail | 83 | 2.42 | 2.20e-10 |
| head | 109 | 2.06 | 3.53e-10 |
| head neuron | 95 | 2.16 | 1.61e-09 |
| cholinergic neuron | 30 | 4.73 | 1.55e-08 |
| GABAergic neuron | 21 | 6.78 | 3.72e-08 |
| motor neuron | 32 | 4.20 | 6.11e-08 |
| Cell | 132 | 1.62 | 2.97e-06 |
| sensory neuron | 36 | 3.19 | 3.18e-06 |
| body region | 137 | 1.59 | 4.40e-06 |
| Anatomy | 138 | 1.58 | 5.36e-06 |
| dorsal nerve cord | 23 | 4.50 | 6.64e-06 |
| body muscle cell | 51 | 2.47 | 7.27e-06 |
| vulval muscle | 39 | 2.82 | 1.77e-05 |
| Sex specific entity | 77 | 1.88 | 8.94e-05 |
| ventral nerve cord | 26 | 3.46 | 1.28e-04 |
| touch receptor neuron | 16 | 5.37 | 1.45e-04 |
| hermaphrodite-specific | 74 | 1.88 | 1.60e-04 |
| non-striated muscle | 56 | 2.12 | 1.97e-04 |
| muscle cell | 57 | 2.01 | 7.30e-04 |
| Organ system | 140 | 1.46 | 7.65e-04 |
| muscular system | 70 | 1.84 | 8.07e-04 |
| Function | 140 | 1.45 | 8.21e-04 |
| body ganglion | 21 | 3.39 | 2.77e-03 |
| HSNR | 12 | 5.64 | 3.12e-03 |
| HSNL | 12 | 5.57 | 3.50e-03 |
| HSN | 12 | 5.57 | 3.50e-03 |
| dorso-rectal ganglion | 11 | 5.89 | 5.35e-03 |
| C. elegans Cell and Anatomy Ontology | 142 | 1.40 | 6.34e-03 |
| VD neuron | 9 | 6.79 | 1.21e-02 |
| VB neuron | 9 | 6.79 | 1.21e-02 |
| dorso-rectal ganglion neuron | 10 | 5.95 | 1.26e-02 |
| lateral nerve cord | 11 | 5.34 | 1.27e-02 |
| serotonergic neuron | 16 | 3.74 | 1.30e-02 |
| VC neuron | 8 | 7.35 | 2.09e-02 |
| organ | 111 | 1.45 | 2.14e-02 |
| VA neuron | 9 | 6.24 | 2.31e-02 |
| interneuron | 23 | 2.70 | 2.98e-02 |
| DVB | 6 | 10.15 | 3.53e-02 |
| DA neuron | 10 | 5.16 | 3.90e-02 |
| sensillum | 34 | 2.15 | 3.90e-02 |
| digestive tract | 94 | 1.49 | 4.02e-02 |
| SDQ | 6 | 9.76 | 4.06e-02 |
| ventral ganglion | 15 | 3.46 | 4.75e-02 |
| head ganglion | 29 | 2.27 | 4.88e-02 |
| lumbar ganglion | 18 | 3.00 | 4.88e-02 |

### GO terms enriched

|  |  |  |
| --- | --- | --- |
| **GO term** | **Number of genes** | **FDR-corrected p-value** |
| synapse | 25 | 5.0e-10 |
| plasma membrane | 42 | 1.9e-07 |
| transmembrane transporter complex | 11 | 3.3e-06 |
| single organism signaling | 12 | 5.5e-05 |
| synaptic vesicle localization | 8 | 6.9e-05 |
| transporter activity | 37 | 1.1e-04 |
| cation channel complex | 8 | 1.3e-04 |
| establishment of vesicle localization | 8 | 1.6e-04 |
| axon | 15 | 1.8e-04 |
| cell communication | 13 | 2.5e-04 |
| regulation of synaptic transmission | 7 | 2.6e-04 |
| gated channel activity | 8 | 8.5e-04 |
| positive regulation of locomotion | 14 | 1.6e-03 |
| eating behavior | 9 | 1.9e-03 |
| cell junction | 17 | 2.0e-03 |
| voltage-gated ion channel activity | 8 | 2.4e-03 |
| regulation of pharyngeal pumping | 7 | 3.7e-03 |
| establishment of localization | 64 | 4.7e-03 |
| regulation of ion transmembrane transport | 6 | 5.3e-03 |
| cation transport | 18 | 9.2e-03 |
| substrate-specific transmembrane transporter activity | 26 | 9.5e-03 |
| regulation of transport | 7 | 1.4e-02 |
| calcium ion transmembrane transport | 5 | 1.6e-02 |
| locomotory behavior | 6 | 1.7e-02 |
| potassium ion transmembrane transport | 8 | 2.1e-02 |
| potassium ion transport | 9 | 2.3e-02 |
| synaptic transmission | 6 | 2.4e-02 |
| vesicle | 11 | 2.5e-02 |
| transmembrane transport | 20 | 2.8e-02 |
| regulation of behavior | 9 | 3.1e-02 |
| mating behavior | 5 | 3.2e-02 |
| secretion | 10 | 3.7e-02 |
| cellular process | 126 | 4.2e-02 |
| synaptic vesicle | 5 | 4.5e-02 |

### Expression clusters enriched

|  |  |  |  |
| --- | --- | --- | --- |
| **Group name** | **Number in cluster** | **Enrichment** | **FDR corrected p** |
| Larval Pan-neural Enriched Genes. | 162 | 4.43 | 1.21e-62 |
| Genes significantly enriched (> 2x, FDR < 5%) in a particular cell-type versus a reference sample of all cells at the same stage. WBPaper00037950:A-class-motor-neurons\_larva\_enriched | 112 | 6.87 | 1.25e-59 |
| WT-Pico Pan-neural Enriched Genes, with genes found multiple times in a single dataset removed (without dups). | 149 | 3.93 | 1.52e-49 |
| Genes significantly enriched (> 2x, FDR < 5%) in a particular cell-type versus a reference sample of all cells at the same stage. WBPaper00037950:glr-1(+)-neurons\_larva\_enriched | 92 | 5.88 | 7.50e-42 |
| Genes significantly enriched (> 2x, FDR < 5%) in a particular cell-type versus a reference sample of all cells at the same stage. WBPaper00037950:all-neurons\_larva\_enriched | 101 | 4.21 | 1.62e-33 |
| Genes predicted to be upregulated more than 2.0 fold in (AFD+AWB) datasets as compared to unsorted whole embryonic cells dataset. | 83 | 4.34 | 1.37e-27 |
| Embryonic Pan-neural Enriched Genes. | 118 | 3.08 | 4.45e-27 |
| Genes enriched in neuronal miRNA-induced silencing complexes (miRISC) as detected by immunoprecipitations and microarray analysis. | 75 | 4.58 | 4.59e-26 |
| Larval A-class motor neuron enriched genes. | 58 | 6.07 | 9.42e-26 |
| Genes significantly enriched (> 2x, FDR < 5%) in a particular cell-type versus a reference sample of all cells at the same stage. WBPaper00037950:AVE-neuron\_embryo\_enriched | 56 | 5.47 | 1.74e-22 |
| Expressed transcripts enriched in embryonic motor neurons (identified by unc-4::GFP expressing cells). | 76 | 3.32 | 9.49e-18 |
| Embryonic A-class motor neuron enriched genes. | 76 | 3.32 | 9.49e-18 |
| Genes downregulated more than 2 fold after 24 hours of AgNPs exposure. | 74 | 2.95 | 1.95e-14 |
| Genes significantly enriched (> 2x, FDR < 5%) in a particular cell-type versus a reference sample of all cells at the same stage. WBPaper00037950:GABAergic-motor-neurons\_embryo\_enriched | 41 | 4.80 | 6.80e-14 |
| Genes significantly enriched (> 2x, FDR < 5%) in a particular cell-type versus a reference sample of all cells at both embryonic and larval stages. WBPaper00037950:A-class-motor-neurons\_CoreEnriched | 23 | 9.17 | 4.35e-13 |
| Genes with expression enriched in PVD and OLL neurons. Data sets were normalized by RMA and transcripts showing relative PVD enrichment (>= 1.5X) vs. the reference sample were identified by SAM analysis (False Discovery Rate, FDR < 1%). | 111 | 2.15 | 6.08e-13 |
| Genes with expression level down regulated in mir-35 mutants comparing with N2. | 69 | 2.88 | 1.02e-12 |
| Genes significantly enriched (> 2x, FDR < 5%) in a particular cell-type versus a reference sample of all cells at the same stage. WBPaper00037950:dopaminergic-neurons\_larva\_enriched | 75 | 2.58 | 1.53e-11 |
| Genes significantly enriched (> 2x, FDR < 5%) in a particular cell-type versus a reference sample of all cells at both embryonic and larval stages. WBPaper00037950:all-neurons\_CoreEnriched | 20 | 8.29 | 2.31e-10 |
| Genes expressed in embryonic motor neurons (identified by unc-4::GFP expressing cells). | 209 | 1.46 | 1.60e-09 |
| mRNAs that were significantly enriched in the AIN-2 immunoprecipitation samples, compared to the control total mRNAs in the input extracts (p < 0.01). | 89 | 2.12 | 2.02e-09 |
| Genes significantly enriched (> 2x, FDR < 5%) in a particular cell-type versus a reference sample of all cells at the same stage. WBPaper00037950:BAG-neuron\_embryo\_enriched | 39 | 3.63 | 2.45e-09 |
| Genes that show selective expression in a subset of cell types vs broadly expressed in many cell types. Correspond to 20% - 57% of enriched\_genes for a given cell type. WBPaper00037950:A-class-motor-neurons\_larva\_SelectivelyEnriched | 30 | 4.51 | 4.07e-09 |
| Genes significantly enriched (> 2x, FDR < 5%) in a particular cell-type versus a reference sample of all cells at the same stage. WBPaper00037950:dopaminergic-neurons\_embryo\_enriched | 39 | 3.54 | 5.22e-09 |
| Genes upregulated by alg-1(-). | 74 | 2.20 | 4.14e-08 |
| Genes that showed higher expression in N2 than in DR1350. | 76 | 2.16 | 4.69e-08 |
| Genes that showed expression levels higher than the corresponding reference sample (embryonic 0hr reference). WBPaper00037950:BAG-neuron\_expressed | 165 | 1.52 | 1.82e-07 |
| Genes that show selective expression in a subset of cell types vs broadly expressed in many cell types. Correspond to 20% - 57% of enriched\_genes for a given cell type. WBPaper00037950:glr-1(+)-neurons\_larva\_SelectivelyEnriched | 25 | 4.11 | 1.36e-06 |
| Genes significantly enriched (> 2x, FDR < 5%) in a particular cell-type versus a reference sample of all cells at the same stage. WBPaper00037950:A-class-motor-neurons\_embryo\_enriched | 31 | 3.36 | 2.19e-06 |
| The first of four clusters of genes up-regulated during L1 arrest. | 23 | 4.21 | 3.57e-06 |
| Genes with increased expression after 24 hours of infection by P.lumniescens Fold changes shown are pathogen vs OP50. WBPaper00038438:P.lumniescens\_24hr\_upregulated\_TilingArray | 137 | 1.53 | 8.45e-06 |
| Genes significantly enriched (> 2x, FDR < 5%) in a particular cell-type versus a reference sample of all cells at the same stage. WBPaper00037950:all-neurons\_embryo\_enriched | 32 | 3.01 | 1.52e-05 |
| Genes depleted in muscle cells (0hr muscle dataset). Dissociated myo-3::GFP embryos were cultured for 0 hours before FACS sorting. | 76 | 1.88 | 1.67e-05 |
| Genes enriched in HLH-1 heat shock dataset. | 94 | 1.71 | 2.49e-05 |
| mRNAs that were significantly enriched in the AIN-1 immunoprecipitation samples, compared to the control total mRNAs in the input extracts (p < 0.01). | 99 | 1.67 | 3.28e-05 |
| Genes differentially expressed under EtBr treatment without UVC exposure vs after UVC exposure but without EtBr treatment at the -3h timepoint (3 h after the third UVC dose (51h), which is also 3 h after being placed on food). | 49 | 2.24 | 4.39e-05 |
| Genes significantly enriched (> 2x, FDR < 5%) in a particular cell-type versus a reference sample of all cells at both embryonic and larval stages. WBPaper00037950:dopaminergic-neurons\_CoreEnriched | 17 | 4.79 | 4.42e-05 |
| Genes that are dosage compensated. | 26 | 2.94 | 3.78e-04 |
| miRNA targets that are significantly enriched at L3 larva stage. To generate a global view of the dynamics of miRNA-mediated regulation of gene expression during C. elegans development, authors analyzed the mRNAs in the AIN-2-GFP IP results from five developmental stages. The magnitude of the combined interaction of miRNAs with a given target mRNA was assessed by measuring the fold enrichment of that mRNA in AIN-2 IP samples, relative to the abundance of the mRNA in the corresponding total lysate. Because this enrichment in the IP sample versus total lysate directly reflects the miRISC-associated fraction of a given mRNA, high enrichment indicates the likelihood of strong miRNA-mediated regulation of the mRNA, whereas low or negative enrichment indicates the likelihood of weak or absent miRNA regulation of the mRNA. It is also possible that poor enrichment could reflect interactions that occur only in a rare subset of cells at any given stage of development. Transcripts that were significantly enriched (0 | 44 | 2.16 | 4.58e-04 |
| Genes that show selective expression in a subset of cell types vs broadly expressed in many cell types. Correspond to 20% - 57% of enriched\_genes for a given cell type. WBPaper00037950:AVE-neuron\_embryo\_SelectivelyEnriched | 15 | 4.47 | 5.20e-04 |
| Total muscle depleted genes (complete list of non-overlapping genes from the 0hr and 24hr muscle depleted datasets). | 94 | 1.60 | 5.21e-04 |
| phasmids (adult); 8.3e-04(11.83x). phasmids (larva); 8.3e-04(11.83x). | 7 | 10.57 | 1.06e-03 |
| miRNA targets that are significantly enriched at L2 larva stage. To generate a global view of the dynamics of miRNA-mediated regulation of gene expression during C. elegans development, authors analyzed the mRNAs in the AIN-2-GFP IP results from five developmental stages. The magnitude of the combined interaction of miRNAs with a given target mRNA was assessed by measuring the fold enrichment of that mRNA in AIN-2 IP samples, relative to the abundance of the mRNA in the corresponding total lysate. Because this enrichment in the IP sample versus total lysate directly reflects the miRISC-associated fraction of a given mRNA, high enrichment indicates the likelihood of strong miRNA-mediated regulation of the mRNA, whereas low or negative enrichment indicates the likelihood of weak or absent miRNA regulation of the mRNA. It is also possible that poor enrichment could reflect interactions that occur only in a rare subset of cells at any given stage of development. Transcripts that were significantly enriched (0 | 42 | 2.13 | 1.06e-03 |
| A complete list of the genes that showed differential expression in a slr-2 mutant strain. | 70 | 1.72 | 1.23e-03 |
| phasmids (adult); 9.4e-04(10.99x). phasmids (larva); 9.4e-04(10.99x). | 7 | 10.20 | 1.34e-03 |
| Genome-wide analysis of developmental and sex-regulated gene expression profile. cgc4489\_group\_14 | 30 | 2.48 | 1.63e-03 |
| phasmids (adult); 1.5e-04(13.99x). phasmids (larva); 1.5e-04(13.99x). dorsal nerve cord (adult); 3.9e-04(7.84x). amphids (adult); 6.0e-04(10.10x). amphids (larva); 6.0e-04(10.10x). | 6 | 12.68 | 1.64e-03 |
| miRNA targets that are significantly enriched at L1 larva stage. To generate a global view of the dynamics of miRNA-mediated regulation of gene expression during C. elegans development, authors analyzed the mRNAs in the AIN-2-GFP IP results from five developmental stages. The magnitude of the combined interaction of miRNAs with a given target mRNA was assessed by measuring the fold enrichment of that mRNA in AIN-2 IP samples, relative to the abundance of the mRNA in the corresponding total lysate. Because this enrichment in the IP sample versus total lysate directly reflects the miRISC-associated fraction of a given mRNA, high enrichment indicates the likelihood of strong miRNA-mediated regulation of the mRNA, whereas low or negative enrichment indicates the likelihood of weak or absent miRNA regulation of the mRNA. It is also possible that poor enrichment could reflect interactions that occur only in a rare subset of cells at any given stage of development. Transcripts that were significantly enriched (0 | 48 | 1.96 | 1.86e-03 |
| Genes that show selective expression in a subset of cell types vs broadly expressed in many cell types. Correspond to 20% - 57% of enriched\_genes for a given cell type. WBPaper00037950:all-neurons\_larva\_SelectivelyEnriched | 31 | 2.40 | 2.08e-03 |
| phasmids (adult); 4.8e-05(17.09x). phasmids (larva); 4.8e-05(17.09x). amphids (adult); 2.0e-04(12.35x). amphids (larva); 2.0e-04(12.35x). dorsal nerve cord (larva); 6.4e-04(9.26x). | 5 | 14.09 | 5.20e-03 |
| Genes significantly enriched (> 2x, FDR < 5%) in a particular cell-type versus a reference sample of all cells at the same stage. WBPaper00037950:AVA-neuron\_embryo\_enriched | 29 | 2.29 | 8.36e-03 |
| miRNA targets that are significantly enriched at L4 larva stage. To generate a global view of the dynamics of miRNA-mediated regulation of gene expression during C. elegans development, authors analyzed the mRNAs in the AIN-2-GFP IP results from five developmental stages. The magnitude of the combined interaction of miRNAs with a given target mRNA was assessed by measuring the fold enrichment of that mRNA in AIN-2 IP samples, relative to the abundance of the mRNA in the corresponding total lysate. Because this enrichment in the IP sample versus total lysate directly reflects the miRISC-associated fraction of a given mRNA, high enrichment indicates the likelihood of strong miRNA-mediated regulation of the mRNA, whereas low or negative enrichment indicates the likelihood of weak or absent miRNA regulation of the mRNA. It is also possible that poor enrichment could reflect interactions that occur only in a rare subset of cells at any given stage of development. Transcripts that were significantly enriched (0 | 37 | 2.03 | 9.16e-03 |
| Genes enriched in body wall muscle AIN-2 miRISCs. Pmyo-3-ain-2-gfp IP was performed in mixed stage worms. | 28 | 2.26 | 1.33e-02 |
| Genes depleted in muscle cells (24hr muscle dataset). Dissociated myo-3::GFP embryos were cultured for 24 hours before FACS sorting. | 62 | 1.65 | 1.35e-02 |
| Genes with variation in expression level across genotypes (AB2, CB4856, RC301, CB4857, N2, HC445). | 73 | 1.56 | 1.50e-02 |
| miRNA targets that are significantly enriched at embryo stage. To generate a global view of the dynamics of miRNA-mediated regulation of gene expression during C. elegans development, authors analyzed the mRNAs in the AIN-2-GFP IP results from five developmental stages. The magnitude of the combined interaction of miRNAs with a given target mRNA was assessed by measuring the fold enrichment of that mRNA in AIN-2 IP samples, relative to the abundance of the mRNA in the corresponding total lysate. Because this enrichment in the IP sample versus total lysate directly reflects the miRISC-associated fraction of a given mRNA, high enrichment indicates the likelihood of strong miRNA-mediated regulation of the mRNA, whereas low or negative enrichment indicates the likelihood of weak or absent miRNA regulation of the mRNA. It is also possible that poor enrichment could reflect interactions that occur only in a rare subset of cells at any given stage of development. Transcripts that were significantly enriched (0 | 35 | 2.01 | 1.78e-02 |
| Genes differentially expressed under EtBr treatment without UVC exposure vs after UVC exposure but without EtBr treatment at the -1h timepoint (just prior to the third UVC dose (48h)). | 105 | 1.41 | 1.83e-02 |
| Genome-wide analysis of developmental and sex-regulated gene expression profile. cgc4489\_group\_13 | 23 | 2.34 | 3.46e-02 |
| Germline-enriched and sex-biased expression profile cluster A. | 30 | 2.04 | 3.96e-02 |
| Genome-wide analysis of developmental and sex-regulated gene expression profile. cgc4489\_group\_19 | 20 | 2.46 | 4.58e-02 |

### Motifs enriched

|  |  |  |  |  |  |
| --- | --- | --- | --- | --- | --- |
| **Motif** | **Logo** | **Possible orthologs** | **Number of motifs in cluster** | **Enrichment** | **FDR corrected p** |
| MA0543.1 |  | eor-1 daf-8 | 219 | 2.24 | 1.4e-35 |
| MA0537.1 |  | blmp-1 | 238 | 1.96 | 9.3e-31 |
| MA0049.1 |  | hbl-1 (0.52) php-3 lin-39 | 185 | 2.25 | 2.1e-27 |
| pTH9930 |  | mel-28 fkh-7 lin-29 | 198 | 2.09 | 4.7e-26 |
| pTH10797 |  | lin-29 K11D2.4 | 191 | 2.12 | 2.1e-25 |
| MA0481.1 |  | fkh-10 (0.64) fkh-8 (0.53) daf-16 let-381 fkh-7 lin-31 | 231 | 1.84 | 5.7e-25 |
| pTH9137 |  | nhr-65 | 225 | 1.87 | 1.5e-24 |
| rn\_SOLEXA\_5\_FBgn0259172 |  | lin-29 | 238 | 1.78 | 7.8e-24 |
| NR2F6\_f1 |  | nhr-239 nhr-2 nhr-62 | 269 | 1.61 | 1.0e-22 |
| pTH9118 |  | ref-2 eor-1 egrh-3 | 185 | 2.06 | 1.1e-22 |
| pTH9242 |  | mel-28 | 174 | 2.14 | 2.1e-22 |
| ZNF75A\_1 |  | F26F4.8 ztf-3 lag-1 | 278 | 1.56 | 3.1e-22 |
| SP1\_f1 |  | klf-1 ZC328.2 klf-2 | 211 | 1.88 | 3.4e-22 |
| SP1\_1 |  | klf-1 klf-2 | 224 | 1.80 | 4.3e-22 |
| MA0452.2 |  | B0310.2 ZK177.3 | 240 | 1.72 | 4.8e-22 |
| PLAG1\_si |  | Y53H1A.2 (0.51) klf-2 plp-2 | 183 | 2.05 | 5.9e-22 |
| Egr3\_1 |  | klf-1 ZC328.2 klf-2 | 201 | 1.92 | 8.6e-22 |
| SPDEF\_3 |  | nhr-100 (0.6) lin-39 lin-1 | 240 | 1.72 | 9.0e-22 |
| pTH9180 |  | mel-28 mef-2 let-381 Y61A9LA.9 Y116A8C.22 | 203 | 1.91 | 9.1e-22 |
| pTH9394 |  | klf-1 sptf-3 klf-2 | 215 | 1.84 | 1.1e-21 |
| Hoxb3\_1720 |  | lin-39 | 222 | 1.80 | 1.4e-21 |
| ELF3\_f1 |  | K02D7.2 unc-120 C24A1.2 | 256 | 1.64 | 1.9e-21 |
| HXD4\_f1 |  | ceh-24 (0.77) ceh-8 (0.66) ceh-9 (0.66) ceh-31 (0.58) ceh-30 ceh-19 alr-1 lim-7 ceh-43 lin-39 cog-1 ceh-1 | 202 | 1.91 | 1.9e-21 |
| pTH9237 |  | mel-28 | 223 | 1.79 | 2.2e-21 |
| FOXJ3\_3 |  | fkh-10 (0.64) fkh-8 (0.53) daf-16 let-381 fkh-7 pha-4 lin-31 | 275 | 1.56 | 3.0e-21 |
| Elf5 |  | C24A1.2 | 243 | 1.69 | 4.0e-21 |
| pTH10629 |  | ceh-9 (0.66) hif-1 (0.63) daf-12 Y5F2A.4 ztf-3 | 248 | 1.66 | 5.4e-21 |
| MA0098.2 |  | lin-1 C24A1.2 | 193 | 1.94 | 7.2e-21 |
| Evx1\_3952 |  | ceh-53 | 207 | 1.85 | 1.1e-20 |
| Gsh2\_3990 |  | ceh-31 (0.58) | 214 | 1.81 | 1.2e-20 |
| Lhx2\_0953 |  | ceh-18 (0.67) lim-4 (0.62) ceh-36 (0.62) ceh-23 (0.59) ceh-31 (0.58) ceh-30 pal-1 lim-7 alr-1 ceh-16 ceh-12 ceh-43 ceh-10 pha-2 ceh-14 ceh-53 mls-2 lin-39 ceh-2 lim-6 and 9 others  [full list] | 131 | 2.50 | 1.6e-20 |
| I$E74A\_01 |  | nhr-19 lin-1 C24A1.2 | 244 | 1.66 | 2.2e-20 |
| Hoxa5\_3415 |  | lin-39 | 216 | 1.78 | 4.7e-20 |
| Hoxd3\_1742 |  | ceh-18 (0.67) npax-3 alr-1 ceh-12 ceh-43 ceh-53 lin-39 ceh-1 ceh-45 | 223 | 1.75 | 5.1e-20 |
| pTH6486 |  | nhr-145 (0.64) | 241 | 1.67 | 5.5e-20 |
| klu\_SOLEXA\_5\_FBgn0013469 |  | ZC328.2 daf-16 | 231 | 1.71 | 5.6e-20 |
| Hoxa2\_3079 |  | lin-39 | 218 | 1.77 | 5.6e-20 |
| Hoxc6\_3954 |  | ceh-18 (0.67) pal-1 alr-1 ceh-12 ceh-43 lin-39 | 195 | 1.90 | 5.9e-20 |
| MA0037.2 |  | ztf-29 elt-1 | 249 | 1.63 | 6.9e-20 |
| exd\_FlyReg\_FBgn0000611 |  | let-381 ceh-20 cfi-1 | 235 | 1.69 | 7.8e-20 |
| Ehf\_3056 |  | lin-1 C24A1.2 | 240 | 1.66 | 1.0e-19 |
| pTH10630 |  | lsy-27 | 228 | 1.72 | 1.1e-19 |
| Lbx2\_3869 |  | mls-2 | 120 | 2.59 | 1.4e-19 |
| pTH8916 |  | ceh-43 ceh-53 let-381 lin-39 hmg-12 lin-31 hmbx-1 Y116A8C.22 | 245 | 1.64 | 1.6e-19 |
| PTF1A\_f1 |  | lin-32 (-0.52) hlh-2 | 251 | 1.61 | 1.9e-19 |
| Zfp691\_0895 |  | F21A9.2 CELE\_Y38H8A.5 | 218 | 1.75 | 2.1e-19 |
| POU4F2\_2 |  | ceh-18 (0.67) unc-86 | 283 | 1.49 | 2.3e-19 |
| pTH6003 |  | nhr-134 nhr-182 | 251 | 1.61 | 2.5e-19 |
| TBX3\_f1 |  | tbx-39 ceh-45 | 223 | 1.73 | 2.9e-19 |
| K562\_SP2\_HudsonAlpha |  | klf-2 | 204 | 1.82 | 3.6e-19 |
| Vax1\_3499 |  | C02F12.10 | 215 | 1.76 | 5.5e-19 |
| FLI1\_f1 |  | lin-1 | 198 | 1.84 | 6.1e-19 |
| Meox1\_2310 |  | ceh-31 (0.58) | 222 | 1.72 | 7.1e-19 |
| SRP000217\_Sox2 |  | sox-4 (0.82) ceh-18 (0.67) ceh-6 tbp-1 | 209 | 1.78 | 7.5e-19 |
| pTH9059 |  | ces-1 che-1 ztf-28 | 224 | 1.71 | 8.0e-19 |
| MA0027.1 |  | ceh-16 | 195 | 1.86 | 8.4e-19 |
| Hoxd9\_2 |  | ceh-24 (0.77) pal-1 php-3 lin-39 | 255 | 1.58 | 8.5e-19 |
| pTH5887 |  | lin-39 | 198 | 1.84 | 8.9e-19 |
| NR2F1\_2 |  | nhr-2 nhr-19 nhr-62 | 194 | 1.85 | 1.2e-18 |
| NR1D1\_f1 |  | nhr-213 nhr-118 | 216 | 1.73 | 1.9e-18 |
| ARNT2\_si |  | aha-1 C46E10.9 | 209 | 1.77 | 2.0e-18 |
| V$XFD1\_01 |  | let-381 lin-31 | 182 | 1.92 | 2.0e-18 |
| pTH5805 |  | alr-1 ceh-10 lin-39 ceh-1 eyg-1 ceh-45 | 198 | 1.82 | 2.2e-18 |
| Alx1\_2 |  | alr-1 ceh-14 cfi-1 ZC204.2 | 250 | 1.59 | 2.5e-18 |
| V$ZID\_01 |  | ceh-32 skn-1 ztf-28 | 211 | 1.75 | 2.8e-18 |
| Elf2 |  | F19F10.1 lin-1 C24A1.2 | 226 | 1.68 | 3.3e-18 |
| MSX2\_2 |  | ceh-9 (0.66) ceh-31 (0.58) ceh-16 lim-7 alr-1 ceh-43 lin-39 ceh-1 | 206 | 1.77 | 3.6e-18 |
| pTH10623 |  | scrt-1 | 224 | 1.69 | 4.1e-18 |
| pTH6268 |  | ceh-2 | 176 | 1.94 | 4.2e-18 |
| TBX1\_1 |  | tbx-38 mab-9 tbx-39 tbx-43 tbx-42 | 189 | 1.86 | 4.8e-18 |
| MAFA\_f1 |  | pax-2 daf-8 F45H11.6 | 233 | 1.65 | 4.8e-18 |
| Ftz\_Cell\_FBgn0001077 |  | php-3 lim-7 lin-39 unc-86 cfi-1 | 205 | 1.77 | 5.2e-18 |
| Mv90 |  | mef-2 | 226 | 1.67 | 6.8e-18 |
| MA0118.1 |  | ref-2 | 183 | 1.89 | 6.8e-18 |
| V$LYF1\_01 |  | mbr-1 (0.86) F26F4.8 bed-3 nhr-177 | 234 | 1.64 | 7.2e-18 |
| HOXC10\_3 |  | ceh-24 (0.77) pal-1 ceh-13 php-3 lin-39 D1005.3 T27F2.4 | 209 | 1.75 | 8.8e-18 |
| MA0139.1 |  | F58G1.2 Y5F2A.4 | 182 | 1.89 | 9.3e-18 |
| Lmx1a\_2238 |  | ceh-24 (0.77) ceh-16 lim-7 lim-6 | 202 | 1.78 | 1.0e-17 |
| pTH9164 |  | ceh-26 | 243 | 1.60 | 1.0e-17 |
| pTH10816 |  | dmd-6 | 212 | 1.73 | 1.1e-17 |
| Hoxa4\_3426 |  | lin-39 | 218 | 1.70 | 1.7e-17 |
| Elf3\_3876 |  | C24A1.2 | 244 | 1.59 | 2.0e-17 |
| Hr51\_SANGER\_5\_FBgn0034012 |  | sox-4 (0.82) nhr-100 (0.6) pop-1 (-0.51) | 245 | 1.58 | 2.2e-17 |
| ARI3A\_do |  | gei-3 (0.76) nhr-208 lim-6 cfi-1 | 226 | 1.66 | 2.2e-17 |
| Nkx6-3\_3446 |  | cog-1 | 198 | 1.79 | 2.3e-17 |
| Vax2\_3500 |  | C02F12.10 | 209 | 1.73 | 2.3e-17 |
| pTH9254 |  | mel-28 | 290 | 1.43 | 2.4e-17 |
| Hoxa3\_2783 |  | lin-39 | 207 | 1.74 | 2.7e-17 |
| ETS2\_f1 |  | lin-1 C24A1.2 | 231 | 1.63 | 2.9e-17 |
| pTH5812 |  | ceh-14 | 196 | 1.79 | 3.4e-17 |
| HeLa-S3\_ZNF274\_UCD |  | C28G1.4 | 224 | 1.66 | 3.4e-17 |
| MA0498.1 |  | lin-32 (-0.52) ceh-32 lin-39 | 227 | 1.65 | 3.8e-17 |
| Hoxb4\_2627 |  | lin-39 | 194 | 1.80 | 3.9e-17 |
| V$TAXCREB\_02 |  | crh-1 zip-3 | 203 | 1.75 | 4.0e-17 |
| ESRRA\_2 |  | nhr-71 (0.59) nhr-6 nhr-2 nhr-213 nhr-10 nhr-68 Y67D8A.3 | 223 | 1.66 | 4.3e-17 |
| pTH9097 |  | Y116A8C.22 | 239 | 1.60 | 5.0e-17 |
| pTH3120 |  | che-1 K11D2.4 tbp-1 | 160 | 2.00 | 6.2e-17 |
| pTH9260 |  | mel-28 | 199 | 1.77 | 6.5e-17 |
| ONEC2\_si |  | ceh-48 (0.64) dsc-1 | 269 | 1.49 | 6.7e-17 |
| pTH2820 |  | ZC328.2 | 204 | 1.74 | 6.9e-17 |
| pTH10633 |  | R07H5.10 C48E7.11 | 202 | 1.75 | 7.1e-17 |
| I$DRI\_01 |  | cfi-1 | 208 | 1.72 | 7.5e-17 |
| pTH3751 |  | tbx-39 | 226 | 1.64 | 8.7e-17 |
| pTH9335 |  | mel-28 | 194 | 1.79 | 8.8e-17 |
| V$VMYB\_01 |  | lin-48 B0310.2 D1081.8 | 209 | 1.71 | 1.0e-16 |
| pTH10038 |  | sox-4 (0.82) gei-3 (0.76) F56D1.1 | 192 | 1.79 | 1.0e-16 |
| Plagl1\_0972 |  | Y53H1A.2 (0.51) | 143 | 2.13 | 1.1e-16 |
| pTH9082 |  | mab-23 | 238 | 1.59 | 1.1e-16 |
| Cart1\_1275 |  | ceh-18 (0.67) alr-1 lim-7 | 195 | 1.78 | 1.2e-16 |
| Etv3 |  | lin-1 | 216 | 1.68 | 1.2e-16 |
| MA0547.1 |  | skn-1 ceh-2 | 211 | 1.70 | 1.3e-16 |
| HXD10\_f1 |  | php-3 nhr-2 | 259 | 1.51 | 1.7e-16 |
| pTH3466 |  | nhr-2 nhr-213 nhr-69 nhr-19 nhr-10 | 188 | 1.81 | 1.8e-16 |
| ATF1\_si |  | crh-1 ceh-26 | 210 | 1.70 | 1.9e-16 |
| Hoxa6\_1040 |  | lin-39 | 221 | 1.65 | 2.0e-16 |
| MEIS1\_1 |  | ces-1 ceh-32 ceh-20 F55C5.11 | 240 | 1.58 | 2.1e-16 |
| V$POU3F2\_01 |  | ceh-18 (0.67) dmd-3 | 259 | 1.51 | 2.1e-16 |
| Emx2\_3420 |  | ceh-2 | 193 | 1.78 | 2.4e-16 |
| MA0249.1 |  | hlh-15 hlh-8 hlh-32 ngn-1 lin-31 | 221 | 1.65 | 2.5e-16 |
| Hoxc4\_3491 |  | lin-39 | 218 | 1.66 | 2.8e-16 |
| pTH5828 |  | nhr-84 (0.52) nhr-216 nhr-142 | 237 | 1.58 | 3.1e-16 |
| Lhx1\_2240 |  | lim-7 | 225 | 1.63 | 3.1e-16 |
| Prop1\_3949 |  | ceh-16 ceh-53 | 178 | 1.85 | 3.1e-16 |
| pTH1001 |  | dnj-17 | 209 | 1.70 | 3.1e-16 |
| Hoxb7\_3953 |  | ceh-18 (0.67) ceh-6 lin-39 | 189 | 1.79 | 3.2e-16 |
| pTH3819 |  | ceh-18 (0.67) | 140 | 2.13 | 3.2e-16 |
| pTH8587 |  | daf-19 (0.85) | 206 | 1.71 | 3.5e-16 |
| pTH9182 |  | tbx-39 | 214 | 1.67 | 3.9e-16 |
| SOX2\_3 |  | sox-4 (0.82) grh-1 dmd-4 | 223 | 1.63 | 3.9e-16 |
| V$TCF11\_01 |  | skn-1 | 229 | 1.61 | 4.0e-16 |
| V$CETS1P54\_02 |  | C52B9.2 | 223 | 1.63 | 4.2e-16 |
| V$FAC1\_01 |  | gei-8 | 266 | 1.48 | 4.6e-16 |
| K562\_ZBTB7A\_HudsonAlpha |  | ZC328.2 | 163 | 1.94 | 4.7e-16 |
| pTH9080 |  | mnm-2 | 212 | 1.68 | 5.1e-16 |
| Elf4 |  | C24A1.2 | 222 | 1.63 | 5.3e-16 |
| pTH6497 |  | lin-31 | 252 | 1.52 | 5.5e-16 |
| pTH9222 |  | mel-28 | 233 | 1.59 | 5.8e-16 |
| Hoxa10\_2318 |  | ceh-24 (0.77) | 237 | 1.57 | 7.7e-16 |
| pTH10647 |  | nhr-232 | 220 | 1.64 | 8.3e-16 |
| Elf3 |  | C24A1.2 | 235 | 1.58 | 8.8e-16 |
| pTH8216 |  | Y116A8C.22 | 209 | 1.68 | 9.0e-16 |
| HXB7\_si |  | ceh-12 lin-39 ceh-20 | 269 | 1.46 | 9.6e-16 |
| pTH9314 |  | atf-6 (0.53) fos-1 crh-1 atf-7 C27D6.4 | 161 | 1.94 | 1.0e-15 |
| Mitf\_SANGER\_5\_FBgn0263112 |  | mdl-1 (0.66) aha-1 pax-1 hlh-30 | 216 | 1.65 | 1.1e-15 |
| pTH1292 |  | ceh-24 (0.77) pzf-1 | 236 | 1.57 | 1.2e-15 |
| pTH10718 |  | egl-43 | 225 | 1.61 | 1.3e-15 |
| vnd\_FlyReg\_FBgn0003986 |  | ceh-24 (0.77) ceh-22 dsc-1 | 246 | 1.53 | 1.5e-15 |
| pTH10041 |  | ztf-29 | 221 | 1.62 | 1.5e-15 |
| FOXO1\_si |  | irx-1 daf-16 fkh-9 | 205 | 1.69 | 1.6e-15 |
| Cdx1\_2245 |  | ceh-13 | 236 | 1.57 | 1.7e-15 |
| Pou2f3\_3986 |  | ceh-18 (0.67) | 237 | 1.56 | 1.8e-15 |
| pTH10823 |  | B0310.2 | 212 | 1.66 | 1.8e-15 |
| pTH8991 |  | cey-3 | 206 | 1.68 | 1.9e-15 |
| pTH9250 |  | dmd-3 C34D1.1 | 239 | 1.55 | 2.1e-15 |
| pTH9323 |  | odr-7 nhr-79 nhr-273 slr-2 nhr-28 lin-1 tbx-39 | 238 | 1.56 | 2.2e-15 |
| KLF8\_f1 |  | klf-1 ZC328.2 | 172 | 1.85 | 2.4e-15 |
| Nkx6-1\_2825 |  | cog-1 | 194 | 1.73 | 2.4e-15 |
| TBP\_f1 |  | tbp-1 | 228 | 1.59 | 2.4e-15 |
| pTH9142 |  | ztf-6 C34D1.1 gei-11 | 240 | 1.55 | 2.6e-15 |
| Foxg1\_2 |  | nhr-213 lin-31 | 212 | 1.65 | 3.6e-15 |
| pTH9244 |  | tbx-39 | 216 | 1.63 | 3.6e-15 |
| Hoxa9\_2622 |  | php-3 lin-39 | 228 | 1.59 | 3.8e-15 |
| V$S8\_01 |  | ceh-45 | 235 | 1.56 | 3.9e-15 |
| pTH9177 |  | F10B5.3 (0.76) hsf-1 Y53C10A.3 | 290 | 1.39 | 3.9e-15 |
| Zic1\_0991 |  | T22C8.4 ref-2 | 166 | 1.88 | 4.1e-15 |
| Dlx3\_1030 |  | ceh-43 | 185 | 1.77 | 4.1e-15 |
| MA0540.1 |  | dpy-27 Y44A6D.3 | 188 | 1.75 | 4.3e-15 |
| GM12878\_SRF\_HudsonAlpha |  | hlh-10 (0.59) unc-120 | 210 | 1.65 | 4.5e-15 |
| pTH6436 |  | ceh-53 | 194 | 1.72 | 4.7e-15 |
| MA0262.1 |  | mab-3 hsf-1 | 259 | 1.48 | 4.7e-15 |
| Tbp\_pr781 |  | tbp-1 | 263 | 1.47 | 4.9e-15 |
| MA0173.1 |  | irx-1 hlh-32 D1081.8 | 233 | 1.56 | 5.0e-15 |
| Hoxa7\_3750 |  | lin-39 | 214 | 1.64 | 5.1e-15 |
| Dlx2\_2273 |  | ceh-43 | 186 | 1.76 | 5.2e-15 |
| Atf1\_3026 |  | crh-1 | 202 | 1.68 | 5.3e-15 |
| V$OCT1\_06 |  | ceh-18 (0.67) ztf-9 (0.61) | 249 | 1.51 | 5.8e-15 |
| pTH5778 |  | egl-5 | 221 | 1.61 | 5.9e-15 |
| Hoxa7\_2668 |  | lin-39 | 204 | 1.67 | 6.1e-15 |
| Vsx1\_1728 |  | alr-1 | 212 | 1.64 | 6.7e-15 |
| Sox1\_2631 |  | sox-4 (0.82) | 257 | 1.48 | 7.7e-15 |
| pTH9216 |  | ceh-18 (0.67) | 206 | 1.66 | 8.4e-15 |
| GATA4\_f1 |  | elt-1 | 230 | 1.57 | 9.2e-15 |
| pTH5914 |  | attf-1 | 160 | 1.90 | 1.0e-14 |
| Irx3\_1 |  | irx-1 | 257 | 1.48 | 1.0e-14 |
| Nkx1-2\_3214 |  | ceh-30 | 190 | 1.73 | 1.2e-14 |
| pTH5260 |  | aha-1 hlh-27 lin-22 hlh-28 | 186 | 1.74 | 1.3e-14 |
| PAX5\_f1 |  | pax-2 pax-3 | 195 | 1.70 | 1.4e-14 |
| HES1\_f1 |  | lin-22 | 201 | 1.67 | 1.7e-14 |
| VENTX\_1 |  | ceh-36 (0.62) alr-1 pha-2 ceh-53 dve-1 ceh-45 | 188 | 1.73 | 1.9e-14 |
| Hoxc11\_3718 |  | ceh-24 (0.77) pal-1 | 235 | 1.54 | 2.0e-14 |
| pTH9900 |  | C46E10.8 C33G8.2 | 238 | 1.53 | 2.0e-14 |
| MA0163.1 |  | Y53H1A.2 (0.51) C09F5.3 D1081.8 | 184 | 1.74 | 2.3e-14 |
| V$CEBP\_01 |  | C48E7.11 | 218 | 1.60 | 2.4e-14 |
| pTH6281 |  | nhr-15 nhr-239 nhr-2 nhr-213 | 206 | 1.64 | 2.4e-14 |
| pTH5099 |  | lin-32 (-0.52) hlh-15 hlh-1 hlh-14 hlh-11 | 279 | 1.40 | 2.8e-14 |
| pTH3998 |  | tbx-39 | 218 | 1.60 | 2.8e-14 |
| pTH5437 |  | ceh-34 | 185 | 1.74 | 2.9e-14 |
| V$OCT1\_03 |  | ceh-18 (0.67) | 212 | 1.62 | 3.0e-14 |
| pTH9384 |  | cfi-1 | 277 | 1.41 | 3.1e-14 |
| MCR\_f1 |  | nhr-255 | 141 | 2.00 | 3.4e-14 |
| MA0007.2 |  | npax-1 (0.52) nhr-255 lin-14 | 226 | 1.56 | 3.7e-14 |
| pTH9958 |  | ztf-2 ztf-6 | 279 | 1.40 | 3.7e-14 |
| pTH10638 |  | dmd-3 C34D1.1 | 190 | 1.71 | 4.2e-14 |
| V$BRN2\_01 |  | ceh-18 (0.67) | 179 | 1.76 | 4.6e-14 |
| Mafk\_3106 |  | F45H11.6 | 236 | 1.53 | 4.9e-14 |
| Pbx1\_3203 |  | ceh-20 | 181 | 1.74 | 5.2e-14 |
| pTH8649 |  | mbr-1 (0.86) | 162 | 1.85 | 5.2e-14 |
| MA0244.1 |  | C48E7.11 | 267 | 1.43 | 5.4e-14 |
| pTH4325 |  | ceh-18 (0.67) | 225 | 1.56 | 5.5e-14 |
| pTH6478 |  | lim-7 | 196 | 1.67 | 5.7e-14 |
| pTH10650 |  | nhr-153 | 164 | 1.83 | 5.8e-14 |
| pTH10654 |  | lsy-27 ceh-90 | 215 | 1.60 | 6.2e-14 |
| PAX5\_si |  | pax-2 D1081.8 F45H11.6 | 213 | 1.60 | 6.9e-14 |
| PRRX1\_f1 |  | alr-1 elt-1 | 311 | 1.31 | 6.9e-14 |
| Hoxb5\_3122 |  | lin-39 | 196 | 1.67 | 7.2e-14 |
| pTH2936 |  | nhr-239 | 204 | 1.64 | 7.6e-14 |
| pTH6562 |  | ceh-5 | 174 | 1.77 | 7.7e-14 |
| pTH9220 |  | mbr-1 (0.86) | 165 | 1.82 | 8.2e-14 |
| pTH6508 |  | nhr-36 (0.74) | 236 | 1.52 | 9.1e-14 |
| Titf1\_1722 |  | ceh-24 (0.77) dsc-1 | 197 | 1.66 | 9.2e-14 |
| ZBTB7A\_1 |  | ZC328.2 pax-3 ref-2 ztf-14 lin-1 | 191 | 1.69 | 9.2e-14 |
| Dlx1\_1741 |  | ceh-43 | 184 | 1.72 | 9.3e-14 |
| I$UBX\_01 |  | lin-39 | 183 | 1.72 | 9.8e-14 |
| Tcf7\_0950 |  | pop-1 (-0.51) | 204 | 1.63 | 1.2e-13 |
| gl\_FlyReg\_FBgn0004618 |  | ceh-24 (0.77) ces-1 C34H4.5 T22H9.4 | 243 | 1.49 | 1.2e-13 |
| pTH6429 |  | F13H6.1 nhr-86 nhr-2 nhr-177 | 187 | 1.70 | 1.3e-13 |
| V$MYOD\_01 |  | ces-1 hlh-2 hlh-15 K02D7.2 hlh-8 hlh-1 | 210 | 1.60 | 1.3e-13 |
| CENPB\_1 |  | F21D5.4 F52B11.1 | 199 | 1.65 | 1.4e-13 |
| Tcf7l2\_3461 |  | pop-1 (-0.51) ceh-20 | 261 | 1.44 | 1.5e-13 |
| V$EN1\_01 |  | ceh-16 atf-2 | 140 | 1.97 | 1.5e-13 |
| pTH9928 |  | jun-1 (0.66) fos-1 crh-1 sknr-1 F45H11.6 | 230 | 1.53 | 1.5e-13 |
| V$CREB\_Q2 |  | crh-1 attf-1 W08E12.1 | 210 | 1.60 | 1.9e-13 |
| NR4A2\_1 |  | nhr-71 (0.59) nhr-6 nhr-43 | 294 | 1.35 | 2.0e-13 |
| pTH6591 |  | lin-31 | 255 | 1.45 | 2.0e-13 |
| pTH8998 |  | daf-19 (0.85) mab-3 | 261 | 1.43 | 2.1e-13 |
| pTH9246 |  | fkh-10 (0.64) let-381 lin-31 C34D1.1 | 240 | 1.50 | 2.2e-13 |
| Pou3f4\_3773 |  | ceh-6 | 144 | 1.93 | 2.4e-13 |
| pTH5111 |  | ref-1 mxl-1 aha-1 mxl-2 hlh-26 lin-22 hlh-30 C27D6.4 | 193 | 1.66 | 2.4e-13 |
| pTH10030 |  | xbp-1 (0.75) C01B12.2 | 185 | 1.70 | 2.4e-13 |
| CG8765\_SANGER\_5\_FBgn0036900 |  | H20J04.3 | 256 | 1.45 | 2.6e-13 |
| pTH3046 |  | Y116A8C.22 | 221 | 1.55 | 2.7e-13 |
| pTH9709 |  | die-1 | 245 | 1.48 | 2.7e-13 |
| pTH5976 |  | irx-1 mxl-1 hlh-30 | 206 | 1.61 | 2.8e-13 |
| pTH5561 |  | nhr-239 | 213 | 1.58 | 3.0e-13 |
| pTH5119 |  | cfi-1 | 236 | 1.50 | 3.3e-13 |
| SMAD3\_f1 |  | daf-8 | 196 | 1.65 | 3.4e-13 |
| Jundm2\_0911 |  | skn-1 fos-1 | 162 | 1.81 | 3.6e-13 |
| pTH2283 |  | odd-2 | 140 | 1.95 | 3.7e-13 |
| pTH9925 |  | nhr-100 (0.6) ztf-11 | 272 | 1.40 | 4.0e-13 |
| pTH10031 |  | mbr-1 (0.86) | 148 | 1.89 | 4.3e-13 |
| V$YY1\_01 |  | lsy-2 | 242 | 1.48 | 4.8e-13 |
| Sox17\_2837 |  | sox-4 (0.82) | 242 | 1.48 | 5.1e-13 |
| srp\_SANGER\_5\_FBgn0003507 |  | ceh-32 ceh-34 egl-27 elt-6 elt-3 elt-7 elt-1 | 210 | 1.58 | 5.4e-13 |
| pTH9135 |  | pop-1 (-0.51) | 243 | 1.48 | 5.6e-13 |
| CREB1\_f1 |  | fos-1 crh-1 atf-5 | 155 | 1.84 | 5.7e-13 |
| CG4854\_SANGER\_10\_FBgn0038766 |  | mxl-1 K11D2.4 | 218 | 1.56 | 5.8e-13 |
| FOXI1\_f1 |  | nfya-2 (0.55) cey-3 dro-1 ceh-20 lin-31 | 240 | 1.48 | 6.5e-13 |
| Pou3f1\_3819 |  | ceh-6 | 217 | 1.56 | 7.2e-13 |
| SOX10\_2 |  | sox-4 (0.82) egl-13 (0.75) K11D2.4 | 261 | 1.42 | 8.5e-13 |
| V$FREAC7\_01 |  | lin-31 | 163 | 1.78 | 8.9e-13 |
| Gata6\_3769 |  | end-3 (0.57) elt-1 | 221 | 1.54 | 9.0e-13 |
| pTH9924 |  | nhr-46 | 256 | 1.43 | 9.1e-13 |
| ELK3\_f1 |  | lin-1 | 186 | 1.67 | 9.5e-13 |
| YMR043W\_831 |  | unc-120 | 220 | 1.54 | 1.0e-12 |
| pTH8996 |  | sma-4 hlh-8 daf-8 | 219 | 1.54 | 1.0e-12 |
| Hoxd1\_3448 |  | ceh-12 | 165 | 1.77 | 1.0e-12 |
| pTH6327 |  | dsc-1 | 202 | 1.60 | 1.0e-12 |
| pTH6556 |  | odd-1 lim-6 | 213 | 1.56 | 1.1e-12 |
| V$CDPCR3\_01 |  | ceh-24 (0.77) ceh-48 (0.64) | 183 | 1.68 | 1.1e-12 |
| pTH10772 |  | ceh-52 | 191 | 1.65 | 1.1e-12 |
| MA0204.1 |  | ceh-32 dmd-5 dmd-4 | 185 | 1.67 | 1.1e-12 |
| pTH3064 |  | crh-1 | 203 | 1.60 | 1.1e-12 |
| Tcf3\_3787 |  | pop-1 (-0.51) | 258 | 1.42 | 1.2e-12 |
| Pou2f1\_3081 |  | ceh-18 (0.67) | 216 | 1.54 | 2.0e-12 |
| V$RFX1\_02 |  | daf-19 (0.85) F52B5.7 | 205 | 1.58 | 2.0e-12 |
| pTH9297 |  | ceh-18 (0.67) | 151 | 1.83 | 2.1e-12 |
| Hoxc8\_3429 |  | lin-39 | 207 | 1.57 | 2.3e-12 |
| disco-r-Cl1\_SANGER\_5\_FBgn0042650 |  | lin-31 nhr-68 F55C5.11 | 196 | 1.61 | 2.4e-12 |
| pTH10013 |  | nhr-168 | 209 | 1.57 | 2.4e-12 |
| EMX1\_2 |  | ceh-16 ceh-2 | 229 | 1.50 | 2.8e-12 |
| HEN1\_si |  | hlh-15 hlh-1 | 189 | 1.64 | 2.9e-12 |
| Nkx1-1\_3856 |  | ceh-30 | 181 | 1.67 | 3.1e-12 |
| Hlxb9\_3422 |  | ceh-12 | 191 | 1.63 | 3.4e-12 |
| pTH5714 |  | nhr-239 | 208 | 1.56 | 3.4e-12 |
| Barx1\_2877 |  | ceh-43 | 167 | 1.73 | 3.6e-12 |
| V$GATA3\_01 |  | elt-1 | 199 | 1.59 | 4.1e-12 |
| pTH10808 |  | ztf-19 | 236 | 1.47 | 4.3e-12 |
| TBX2\_f1 |  | ztf-6 tbx-39 | 254 | 1.42 | 5.1e-12 |
| pTH10656 |  | hlh-15 hlh-12 hlh-8 hlh-32 ngn-1 | 222 | 1.51 | 5.1e-12 |
| Gata5\_3768 |  | elt-1 | 217 | 1.53 | 5.1e-12 |
| V$CDXA\_01 |  | ceh-13 php-3 | 210 | 1.55 | 5.5e-12 |
| pTH6106 |  | nhr-182 | 186 | 1.64 | 5.6e-12 |
| pTH10034 |  | nhr-66 | 199 | 1.59 | 5.9e-12 |
| pTH9261 |  | dmd-3 lin-48 pax-3 | 288 | 1.33 | 6.1e-12 |
| pTH9951 |  | pal-1 mex-6 | 178 | 1.67 | 6.3e-12 |
| pTH6449 |  | ceh-43 | 191 | 1.62 | 6.4e-12 |
| LHX9\_2 |  | ceh-31 (0.58) ceh-43 ceh-14 lin-39 ceh-1 | 233 | 1.48 | 6.5e-12 |
| pTH9163 |  | nhr-3 (0.6) | 231 | 1.48 | 7.3e-12 |
| MA0095.2 |  | lsy-2 | 219 | 1.52 | 7.5e-12 |
| Hoxd13\_2356 |  | pal-1 | 248 | 1.43 | 8.1e-12 |
| pTH6445 |  | ceh-5 | 231 | 1.48 | 8.7e-12 |
| pTH7875 |  | mel-28 | 236 | 1.46 | 9.0e-12 |
| pTH8411 |  | tbx-39 | 220 | 1.51 | 9.3e-12 |
| Cdx2\_4272 |  | ceh-13 | 229 | 1.48 | 1.1e-11 |
| V$GR\_Q6 |  | nhr-255 | 224 | 1.49 | 1.2e-11 |
| Gata3\_1024 |  | elt-1 | 213 | 1.53 | 1.4e-11 |
| Irx6\_2623 |  | irx-1 | 181 | 1.64 | 1.5e-11 |
| pTH9108 |  | daf-12 nhr-5 | 267 | 1.38 | 1.5e-11 |
| Mw140 |  | efl-1 F49E12.6 | 245 | 1.43 | 1.6e-11 |
| pTH9198 |  | dmd-3 | 293 | 1.31 | 1.8e-11 |
| pTH6641 |  | lin-31 | 241 | 1.44 | 2.0e-11 |
| Srf\_3509 |  | unc-120 | 205 | 1.55 | 2.0e-11 |
| pTH5118 |  | cfi-1 | 229 | 1.47 | 2.1e-11 |
| pTH9245 |  | ceh-18 (0.67) | 231 | 1.47 | 2.3e-11 |
| CUX1\_2 |  | ceh-48 (0.64) | 244 | 1.43 | 2.4e-11 |
| pTH8983 |  | tag-347 | 202 | 1.56 | 2.4e-11 |
| HIF1A\_si |  | hif-1 (0.63) | 176 | 1.66 | 2.5e-11 |
| pTH9381 |  | ceh-18 (0.67) | 226 | 1.48 | 3.0e-11 |
| pTH10027 |  | M03D4.4 | 173 | 1.67 | 3.1e-11 |
| pTH6636 |  | egl-5 | 174 | 1.66 | 3.2e-11 |
| Pou3f3\_3235 |  | ceh-6 | 137 | 1.86 | 3.3e-11 |
| pTH6569 |  | ceh-43 | 186 | 1.61 | 3.3e-11 |
| pTH5922 |  | ceh-24 (0.77) | 171 | 1.67 | 3.7e-11 |
| Hey\_SANGER\_5\_FBgn0027788 |  | lin-22 gei-11 | 169 | 1.68 | 3.9e-11 |
| pTH9043 |  | sem-2 | 214 | 1.51 | 4.2e-11 |
| Bsx\_3483 |  | ceh-31 (0.58) | 161 | 1.71 | 4.3e-11 |
| pTH9907 |  | nhr-34 | 264 | 1.37 | 4.3e-11 |
| pTH2846 |  | lin-31 | 266 | 1.37 | 5.0e-11 |
| pTH9262 |  | lin-54 | 142 | 1.81 | 5.6e-11 |
| pTH9322 |  | nhr-100 (0.6) nhr-10 nhr-7 elt-1 | 330 | 1.22 | 6.2e-11 |
| Mw160 |  | nhr-68 | 222 | 1.48 | 6.3e-11 |
| pTH5924 |  | nhr-255 | 182 | 1.61 | 6.8e-11 |
| pTH9974 |  | hlh-32 ngn-1 hlh-16 | 269 | 1.36 | 7.0e-11 |
| Pknox2\_3077 |  | ceh-32 | 195 | 1.56 | 7.3e-11 |
| pTH9026 |  | attf-1 | 148 | 1.77 | 7.4e-11 |
| Barx2\_3447 |  | ceh-43 | 197 | 1.55 | 8.8e-11 |
| pTH3997 |  | C04F5.9 | 160 | 1.70 | 8.9e-11 |
| pTH6482 |  | ceh-19 | 185 | 1.60 | 9.1e-11 |
| pTH2684 |  | fos-1 | 309 | 1.26 | 9.6e-11 |
| Tcf1\_2666 |  | hmbx-1 | 158 | 1.71 | 1.1e-10 |
| V$PAX2\_02 |  | pax-1 | 211 | 1.50 | 1.1e-10 |
| Sox8\_1733 |  | sox-4 (0.82) gei-3 (0.76) pop-1 (-0.51) C05C9.3 | 175 | 1.63 | 1.2e-10 |
| pTH8671 |  | attf-1 C01B12.2 | 150 | 1.74 | 1.4e-10 |
| V$GATA6\_01 |  | elt-1 | 214 | 1.49 | 1.4e-10 |
| pTH8985 |  | athp-1 | 195 | 1.55 | 1.5e-10 |
| pTH9387 |  | C34D1.1 | 155 | 1.72 | 1.7e-10 |
| pTH3041 |  | atf-2 | 171 | 1.64 | 2.0e-10 |
| YY1\_1 |  | lsy-2 | 180 | 1.60 | 2.0e-10 |
| TCF4\_2 |  | hlh-2 ztf-6 | 195 | 1.55 | 2.2e-10 |
| Mv75 |  | elt-1 lin-31 | 184 | 1.59 | 2.2e-10 |
| Tcf1\_2666 |  | hmbx-1 | 157 | 1.70 | 2.2e-10 |
| pTH9256 |  | ceh-18 (0.67) | 200 | 1.53 | 2.5e-10 |
| pTH9326 |  | nhr-122 (-0.65) | 217 | 1.47 | 2.5e-10 |
| pTH8318 |  | attf-1 | 110 | 2.01 | 2.6e-10 |
| pTH3477 |  | daf-16 | 259 | 1.36 | 2.8e-10 |
| pTH3831 |  | ces-2 (-0.53) atf-2 C48E7.11 F23F12.9 Y51H4A.4 C01B12.2 | 330 | 1.21 | 3.2e-10 |
| pTH10777 |  | dmd-3 | 282 | 1.31 | 3.6e-10 |
| pTH9353 |  | ceh-51 | 192 | 1.55 | 3.6e-10 |
| Hoxb8\_3780 |  | lin-39 | 166 | 1.65 | 3.8e-10 |
| Hoxa11\_2218 |  | php-3 | 209 | 1.49 | 3.9e-10 |
| pTH9085 |  | nhr-42 | 177 | 1.60 | 4.0e-10 |
| Hmx3\_3490 |  | ceh-9 (0.66) | 207 | 1.50 | 4.7e-10 |
| Six1\_0935 |  | ceh-32 | 282 | 1.31 | 4.7e-10 |
| V$HOX13\_01 |  | lin-39 | 202 | 1.51 | 4.9e-10 |
| pTH9149 |  | ztf-30 | 136 | 1.79 | 5.5e-10 |
| MA0131.1 |  | F39B2.1 ZC416.1 | 170 | 1.62 | 6.2e-10 |
| pTH2280 |  | mnm-2 | 156 | 1.68 | 6.9e-10 |
| Irx2\_0900 |  | irx-1 | 159 | 1.66 | 8.0e-10 |
| Mf28 |  | elt-1 | 218 | 1.46 | 8.3e-10 |
| pTH1014 |  | atf-5 | 167 | 1.63 | 9.1e-10 |
| pTH9969 |  | pag-3 | 238 | 1.40 | 9.2e-10 |
| T-47D\_GATA3\_HudsonAlpha |  | elt-1 | 224 | 1.44 | 1.0e-09 |
| MA0161.1 |  | nfi-1 F49E12.6 | 200 | 1.50 | 1.3e-09 |
| Sox15\_3457 |  | sox-4 (0.82) | 232 | 1.41 | 1.3e-09 |
| HXA7\_f1 |  | lin-39 elt-1 | 203 | 1.49 | 1.5e-09 |
| pTH9215 |  | C34D1.1 | 200 | 1.50 | 1.8e-09 |
| pTH5078 |  | ces-2 (-0.53) | 174 | 1.58 | 2.2e-09 |
| pTH8679 |  | pax-2 | 172 | 1.59 | 2.3e-09 |
| pTH8745 |  | attf-1 | 143 | 1.72 | 2.4e-09 |
| pTH9934 |  | Y53H1A.2 (0.51) | 159 | 1.64 | 2.4e-09 |
| pTH1049 |  | elt-1 | 202 | 1.48 | 2.6e-09 |
| Hnf4a\_2640 |  | nhr-62 | 169 | 1.60 | 2.7e-09 |
| V$IK2\_01 |  | F26F4.8 | 194 | 1.51 | 2.8e-09 |
| pTH9708 |  | ceh-34 | 294 | 1.27 | 2.9e-09 |
| Spdef |  | lin-1 | 191 | 1.51 | 3.5e-09 |
| pTH6447 |  | ceh-19 | 198 | 1.49 | 3.6e-09 |
| tgo\_ss\_SANGER\_5\_FBgn0015014 |  | aha-1 ahr-1 | 178 | 1.56 | 3.9e-09 |
| Poxm\_SOLEXA\_5\_FBgn0003129 |  | pax-2 | 290 | 1.27 | 5.1e-09 |
| pTH10788 |  | tbx-33 | 262 | 1.33 | 5.4e-09 |
| pTH10769 |  | Y48G1C.6 | 182 | 1.53 | 5.7e-09 |
| Tcfap2a\_2337 |  | aptf-1 F58G1.2 | 110 | 1.90 | 6.7e-09 |
| pTH7032 |  | F52B11.1 | 149 | 1.66 | 7.9e-09 |
| pTH8982 |  | ceh-48 (0.64) | 140 | 1.70 | 8.5e-09 |
| Six2\_2307 |  | ceh-32 ceh-34 | 271 | 1.30 | 8.9e-09 |
| pTH9165 |  | ztf-27 | 191 | 1.50 | 1.0e-08 |
| MA0454.1 |  | odd-2 odd-1 | 166 | 1.58 | 1.2e-08 |
| Smad3\_3805 |  | daf-8 | 193 | 1.49 | 1.2e-08 |
| pTH8556 |  | pax-2 | 157 | 1.61 | 1.4e-08 |
| Gmeb1\_1745 |  | attf-1 | 132 | 1.73 | 1.5e-08 |
| V$NCX\_01 |  | ceh-19 | 176 | 1.54 | 1.6e-08 |
| pTH9173 |  | efl-2 | 155 | 1.61 | 2.2e-08 |
| HepG2\_HSF1\_Stanford |  | Y53C10A.3 | 176 | 1.53 | 2.4e-08 |
| pTH10768 |  | med-2 (0.59) | 285 | 1.27 | 2.6e-08 |
| pTH5690 |  | ceh-32 | 164 | 1.57 | 2.9e-08 |
| pTH5919 |  | irx-1 | 285 | 1.26 | 3.0e-08 |
| Zfp161\_2858 |  | pzf-1 | 153 | 1.61 | 3.1e-08 |
| Irx5\_2385 |  | irx-1 | 180 | 1.51 | 3.3e-08 |
| pTH8863 |  | hmg-12 | 212 | 1.41 | 4.4e-08 |
| pTH9880 |  | end-1 | 178 | 1.51 | 5.7e-08 |
| pTH5250 |  | C48E7.11 | 217 | 1.40 | 5.7e-08 |
| Spdef\_0905 |  | lin-1 | 172 | 1.52 | 5.7e-08 |
| V$AREB6\_02 |  | ztf-6 | 187 | 1.47 | 8.4e-08 |
| pTH6423 |  | pha-2 | 183 | 1.48 | 9.3e-08 |
| TLX1\_f1 |  | ceh-19 | 171 | 1.52 | 1.0e-07 |
| pTH5916 |  | efl-2 | 156 | 1.56 | 1.6e-07 |
| pTH1294 |  | mel-28 | 168 | 1.51 | 1.9e-07 |
| pTH10028 |  | nhr-204 | 230 | 1.35 | 1.9e-07 |
| GRHL1\_2 |  | grh-1 | 283 | 1.25 | 2.3e-07 |
| I$DFD\_01 |  | lin-39 | 174 | 1.49 | 2.7e-07 |
| BARHL2\_1 |  | ceh-31 (0.58) | 101 | 1.84 | 2.8e-07 |
| Irx3\_0920 |  | irx-1 | 144 | 1.58 | 4.2e-07 |
| Six4\_2860 |  | ceh-32 | 144 | 1.58 | 4.9e-07 |
| Cutl1\_3494 |  | ceh-44 (0.59) | 164 | 1.51 | 5.4e-07 |
| pTH5781 |  | ceh-32 | 328 | 1.16 | 5.5e-07 |
| pTH10805 |  | ztf-16 | 310 | 1.19 | 7.1e-07 |
| V$GATA1\_02 |  | elt-1 | 174 | 1.47 | 7.8e-07 |
| I$MTTFA\_01 |  | hmg-5 nsy-7 | 189 | 1.42 | 8.1e-07 |
| Six3\_1732 |  | ceh-34 | 275 | 1.25 | 9.6e-07 |
| pTH9096 |  | T07C12.11 | 139 | 1.57 | 1.5e-06 |
| V$GATA1\_01 |  | elt-1 | 263 | 1.26 | 2.0e-06 |
| MA0032.1 |  | let-381 | 127 | 1.61 | 2.3e-06 |
| pTH5891 |  | nhr-49 | 163 | 1.48 | 2.5e-06 |
| V$MYB\_Q6 |  | D1081.8 | 146 | 1.53 | 2.9e-06 |
| pTH10798 |  | Y75B8A.6 | 169 | 1.45 | 3.3e-06 |
| pTH5257 |  | C48E7.11 | 190 | 1.39 | 3.7e-06 |
| pTH9306 |  | syd-9 lsy-2 lsl-1 | 305 | 1.19 | 4.4e-06 |
| pTH5928 |  | ceh-34 | 265 | 1.24 | 1.2e-05 |
| pTH9076 |  | C01G12.1 | 214 | 1.32 | 2.0e-05 |
| Hmbox1\_2674 |  | hmbx-1 | 288 | 1.19 | 3.3e-05 |
| pTH2933 |  | F58G1.2 | 100 | 1.52 | 1.0e-03 |

### Correlated (and anti-correlated) transcription factors

|  |  |
| --- | --- |
| **Transcription factor** | **Correlation** |
| ctbp-1 | 0.95 |
| daf-3 | 0.91 |
| ceh-62 | 0.90 |
| mgl-2 | 0.87 |
| zfh-2 | 0.86 |
| aptf-4 | 0.86 |
| mbr-1 | 0.86 |
| daf-19 | 0.85 |
| nhr-95 | 0.85 |
| madf-4 | 0.84 |
| F26A10.2 | 0.84 |
| unc-42 | 0.84 |
| saeg-2 | 0.83 |
| nhr-1 | 0.83 |
| tag-97 | 0.83 |
| sox-4 | 0.82 |
| dmd-10 | 0.82 |
| ceh-54 | 0.82 |
| sem-4 | 0.82 |
| C34F6.9 | 0.80 |
| C02F5.12 | 0.80 |
| nhr-17 | 0.78 |
| ceh-58 | 0.78 |
| nhr-158 | 0.78 |
| ceh-24 | 0.77 |
| ztf-13 | -0.44 |
| nhr-210 | -0.45 |
| C30G4.7 | -0.45 |
| sma-4 | -0.46 |
| dmd-3 | -0.47 |
| hmg-5 | -0.47 |
| ces-1 | -0.48 |
| sex-1 | -0.48 |
| ceh-82 | -0.48 |
| hmg-6 | -0.49 |
| pop-1 | -0.51 |
| lin-32 | -0.52 |
| nhr-229 | -0.52 |
| C16A3.4 | -0.53 |
| nhr-64 | -0.53 |
| ces-2 | -0.53 |
| icd-2 | -0.55 |
| hlh-29 | -0.55 |
| F27D4.4 | -0.56 |
| Y53F4B.3 | -0.57 |
| zip-7 | -0.58 |
| nhr-13 | -0.59 |
| sup-35 | -0.59 |
| duxl-1 | -0.61 |
| nhr-122 | -0.65 |

### ChIP peaks enriched

|  |  |  |  |  |
| --- | --- | --- | --- | --- |
| **Gene** | **Experiment** | **Number of upstream peaks** | **Enrichment** | **FDR corrected p** |
| ces-1 | CES-1\_Embryos | 230 | 2.60 | 3.9e-52 |
| ces-1 | CES-1\_Fed-L1-stage-larvae | 140 | 4.11 | 1.3e-47 |
| zag-1 | ZAG-1\_Larvae-L2-stage | 161 | 2.98 | 2.5e-38 |
| ces-1 | CES-1\_Larvae-L3-stage | 112 | 3.48 | 5.1e-30 |
| alr-1 | ALR-1\_Larvae-L2-stage | 107 | 3.45 | 3.8e-28 |
| F45C12.2 | F45C12.2\_Fed-L1-stage-larvae | 94 | 3.52 | 6.7e-25 |
| C34F6.9 | C34F6.9\_Larvae-L2-stage | 175 | 2.03 | 1.7e-21 |
| unc-62 | UNC-62\_Larvae-L3-stage | 125 | 2.53 | 2.2e-21 |
| ces-1 | CES-1\_Larvae-L4-stage | 95 | 2.95 | 8.5e-20 |
| sem-4 | SEM-4\_Larvae-L2-stage | 148 | 2.15 | 1.9e-19 |
| lin-35 | LIN-35\_Fed-L1-stage-larvae | 86 | 3.12 | 4.0e-19 |
| ham-1 | HAM-1\_Larvae-L4-stage | 145 | 2.01 | 2.8e-16 |
| zag-1 | ZAG-1\_Larvae-L3-stage | 53 | 4.05 | 1.1e-15 |
| lsy-2 | LSY-2\_Larvae-L1-stage | 103 | 2.42 | 1.4e-15 |
| sax-3 | SAX-3\_Larvae-L2-stage | 110 | 2.32 | 1.5e-15 |
| nfya-1 | NFYA-1\_Larvae-L3-stage | 69 | 3.19 | 2.0e-15 |
| zag-1 | ZAG-1\_Fed-L1-stage-larvae | 70 | 3.12 | 3.3e-15 |
| sax-3 | SAX-3\_Larvae-L4-stage | 99 | 2.42 | 7.6e-15 |
| eor-1 | EOR-1\_Larvae-L3-stage | 137 | 1.94 | 6.9e-14 |
| gei-11 | GEI-11\_Larvae-L2-stage | 62 | 3.15 | 1.6e-13 |
| gei-11 | GEI-11\_Larvae-L3-stage | 73 | 2.79 | 2.0e-13 |
| egl-5 | EGL-5\_Larvae-L3-stage | 122 | 2.02 | 4.2e-13 |
| ceh-26 | CEH-26\_Late-Embryonic-stage | 115 | 2.06 | 8.2e-13 |
| mab-5 | MAB-5\_Larvae-L2-stage | 52 | 3.31 | 6.1e-12 |
| sea-2 | SEA-2\_Larvae-L3-stage | 61 | 2.92 | 7.2e-12 |
| W03F9.2 | W03F9.2\_L4-Young-Adult-stage-larvae | 91 | 2.26 | 7.6e-12 |
| nfya-1 | NFYA-1\_Late-Embryos | 76 | 2.46 | 2.2e-11 |
| pes-1 | PES-1\_Larvae-L4-stage | 76 | 2.46 | 2.3e-11 |
| gei-11 | GEI-11\_Fed-L1-stage-larvae | 65 | 2.67 | 5.0e-11 |
| ham-1 | HAM-1\_Fed-L1-stage-larvae | 74 | 2.46 | 5.1e-11 |
| fos-1 | FOS-1\_Fed-L1-stage-larvae | 71 | 2.52 | 5.2e-11 |
| lin-13 | LIN-13\_Larvae-L2-stage | 47 | 3.23 | 2.3e-10 |
| lsy-2 | LSY-2\_Fed-L1-stage-larvae | 72 | 2.39 | 4.3e-10 |
| nhr-6 | NHR-6\_Larvae-L2-stage | 112 | 1.89 | 5.2e-10 |
| mef-2 | MEF-2\_Fed-L1-stage-larvae | 47 | 3.12 | 6.9e-10 |
| nhr-129 | NHR-129\_Larvae-L2-stage | 177 | 1.53 | 1.9e-09 |
| dpl-1 | DPL-1\_Larvae-L4-stage | 83 | 2.09 | 6.2e-09 |
| efl-1 | EFL-1\_Larvae-L1-stage | 62 | 2.41 | 9.8e-09 |
| unc-62 | UNC-62\_Larvae-L2-stage | 64 | 2.22 | 1.1e-07 |
| zag-1 | ZAG-1\_Larvae-L4-stage | 89 | 1.89 | 1.6e-07 |
| elt-1 | ELT-1\_Larvae-L3-stage | 68 | 2.13 | 1.7e-07 |
| F45C12.2 | F45C12.2\_Larvae-L3-stage | 36 | 2.97 | 5.9e-07 |
| fkh-2 | FKH-2\_Larvae-L3-stage | 65 | 2.07 | 1.2e-06 |
| unc-39 | UNC-39\_Larvae-L2-stage | 33 | 2.87 | 5.0e-06 |
| ceh-38 | CEH-38\_Larvae-L4-stage | 74 | 1.86 | 7.1e-06 |
| efl-1 | EFL-1\_Fed-L1-stage-larvae | 41 | 2.42 | 1.2e-05 |
| sax-3 | SAX-3\_Larvae-L3-stage | 62 | 1.91 | 3.4e-05 |
| ztf-4 | ZTF-4\_Larvae-L2-stage | 25 | 3.00 | 7.6e-05 |
| sax-3 | SAX-3\_Fed-L1-stage-larvae | 53 | 1.99 | 8.1e-05 |
| lsy-2 | LSY-2\_Larvae-L4-stage | 32 | 2.50 | 1.3e-04 |
| hlh-30 | HLH-30\_Larvae-L4-stage | 36 | 2.34 | 1.3e-04 |
| jun-1 | JUN-1\_Larvae-L1-stage | 47 | 2.05 | 1.5e-04 |
| nhr-77 | NHR-77\_Fed-L1-stage-larvae | 57 | 1.86 | 2.2e-04 |
| lsy-2 | LSY-2\_Embryos | 37 | 2.24 | 2.6e-04 |
| R02D3.7 | R02D3.7\_Larvae-L3-stage | 63 | 1.76 | 3.4e-04 |
| fos-1 | FOS-1\_Larvae-L2-stage | 67 | 1.72 | 4.1e-04 |
| F45C12.2 | F45C12.2\_Larvae-L2-stage | 46 | 1.91 | 1.0e-03 |
| aly-2 | ALY-2\_Fed-L1-stage-larvae | 37 | 2.05 | 1.5e-03 |
| lin-15 | LIN-15B\_Fed-L1-stage-larvae | 28 | 2.29 | 2.2e-03 |
| fos-1 | FOS-1\_Larvae-L3-stage | 13 | 3.66 | 3.0e-03 |
| daf-12 | DAF-12\_Larvae-L3-stage | 30 | 2.14 | 3.8e-03 |
| hlh-30 | HLH-30\_Late-Embryos | 56 | 1.63 | 7.5e-03 |
| sma-9 | SMA-9\_Larvae-L2-stage | 22 | 2.33 | 9.2e-03 |
| aly-2 | ALY-2\_Larvae-L2-stage | 18 | 2.49 | 1.5e-02 |
| lsy-2 | LSY-2\_Larvae-L2-stage | 20 | 2.29 | 2.0e-02 |
| fos-1 | FOS-1\_Larvae-L4-stage | 52 | 1.59 | 2.1e-02 |
| nhr-237 | NHR-237\_Embryos | 21 | 2.18 | 2.7e-02 |
| nhr-11 | NHR-11\_Larvae-L2-stage | 48 | 1.58 | 3.5e-02 |
| aly-2 | ALY-2\_Larvae-L3-stage | 7 | 4.27 | 4.3e-02 |
